# Circadian disruption with constant light exposure exacerbates atherosclerosis in male *ApolipoproteinE*-deficient mice

**DOI:** 10.1038/s41598-020-66834-9

**Published:** 2020-06-18

**Authors:** Jeffrey M. Chalfant, Deborah A. Howatt, Lisa R. Tannock, Alan Daugherty, Julie S. Pendergast

**Affiliations:** 1 Department of Biology, University of Kentucky, Lexington, Kentucky USA; 2 Saha Cardiovascular Research Center, University of Kentucky, Lexington, Kentucky USA; 3 Department of Veterans Affairs, Lexington, Kentucky USA; 4 Department of Internal Medicine, University of Kentucky, Lexington, Kentucky USA; 5 Barnstable Brown Diabetes Center, University of Kentucky, Lexington, Kentucky USA; 6 Department of Physiology, University of Kentucky, Lexington, Kentucky USA

**Keywords:** Atherosclerosis, Circadian mechanisms

## Abstract

Disruption of the circadian system caused by disordered exposure to light is pervasive in modern society and increases the risk of cardiovascular disease. The mechanisms by which this happens are largely unknown. ApolipoproteinE-deficient *(ApoE*^−/−^) mice are studied commonly to elucidate mechanisms of atherosclerosis. In this study, we determined the effects of light-induced circadian disruption on atherosclerosis in *ApoE*^−/−^ mice. We first characterized circadian rhythms of behavior, light responsiveness, and molecular timekeeping in tissues from *ApoE*^−/−^ mice that were indistinguishable from rhythms in *ApoE*^+/+^ mice. These data showed that *ApoE*^−/−^ mice had no inherent circadian disruption and therefore were an appropriate model for our study. We next induced severe disruption of circadian rhythms by exposing *ApoE*^−/−^ mice to constant light for 12 weeks. Constant light exposure exacerbated atherosclerosis in male, but not female, *ApoE*^−/−^ mice. Male *ApoE*^−/−^ mice exposed to constant light had increased serum cholesterol concentrations due to increased VLDL/LDL fractions. Taken together, these data suggest that *ApoE*^−/−^ mice are an appropriate model for studying light-induced circadian disruption and that exacerbated dyslipidemia may mediate atherosclerotic lesion formation caused by constant light exposure.

## Introduction

More than 600,000 people die every year in the U.S. from heart disease^1^. Atherosclerosis, the progressive accumulation of plaques in arteries, is the primary cause of cardiovascular disease (CVD), stroke, and myocardial infarction^2^. There are several well-established CVD risk factors including smoking, hypertension, elevated low-density lipoprotein (LDL) cholesterol, obesity, diabetes, and inflammation^3–5^. Epidemiological and clinical studies have shown that circadian disruption also increases the risk of CVD^6–8^.

Circadian rhythms are 24-hour oscillations in gene expression, physiology, and behavior. These rhythms are entrained, or synchronized, with environmental cycles. This allows organisms to anticipate predictable daily changes in the environment to increase their fitness^9–11^. Mammalian circadian rhythms are generated by molecular clocks that are located in nearly every tissue in the body^12^. These clocks are organized hierarchically. The retina detects and sends information about the light-dark cycle to the master clock in the suprachiasmatic nucleus (SCN). The SCN, in turn, coordinates the timing of clocks located throughout the body^12^. At the molecular level, the timekeeping mechanism is a negative transcription/translation feedback loop^13^. The transcription factors BMAL1 and CLOCK dimerize and drive the transcription of the *Period* and *Cryptochrome* genes. Then PERIOD and CRYPTOCHROME feed back and inhibit the transcription factor activity of BMAL1 and CLOCK, thereby suppressing their own transcription. This feedback loop takes approximately 24-hour to complete and therefore generates a 24-hour molecular rhythm.

Recent meta-analyses show that shift workers, who experience chronic circadian disruption and disordered exposure to light and food intake, have 20–40% increased risk of CVD^6,8^. Laboratory studies have also demonstrated a causal link between disruption of circadian rhythms and CVD risk factors. In a controlled laboratory study where young, healthy adults were exposed to a shift work-like protocol for 8 days, participants had increased blood pressure and adverse inflammation, which are associated with CVD^14^. Despite the demonstrated link between circadian disruption and CVD, the underlying mechanisms are largely unknown. The goal of this study was to establish a mouse model for studying the mechanisms of light-induced circadian disruption on atherosclerosis.

Previous studies in mice have shown that genetic disruption of the circadian molecular timekeeping mechanism causes cardiovascular pathology^15–19^. Mice lacking functional *Bmal1*, and *Clock*^*∆19*^ mutants, both of which have impaired or arrhythmic circadian clocks, had increased pathological vascular remodeling compared to mice with functional clocks^17^. *Bmal1*^−/−^ or *Clock*^*∆19*^ mice, on ApolipoproteinE-deficient (*ApoE*^−/−^) or Low-density lipoprotein receptor deficient (*Ldlr*^−/−^) backgrounds, had dysfunctional cholesterol metabolism and transport and increased atherosclerosis^15,16^. In addition, deletion of *Bmal1* in myeloid cells or *Rev-erbα* in bone marrow-derived cells increased atherosclerosis in mice, demonstrating a role of circadian clocks in the immune system in regulating atherosclerosis^18,20^. However, one study showed that inducible deletion of *Bmal1* in adulthood did not increase atherosclerosis, suggesting that the detrimental effects of BMAL1 deficiency may be developmental^19^.

Overall, studies of mutant mice have provided proof of principle that circadian gene mutations increase atherosclerosis. A few studies have also shown that genetic variations of circadian genes are associated with CVD risk factors in humans and circadian gene expression is altered in the cells in the vascular wall by atherosclerosis^21–26^. However, many more studies have shown that disruption of circadian rhythms via environmental disruptors, such as disordered exposure to light and food during shift work, exposure to artificial light at night, and late-night snacking, increases the risk for CVD in humans^8,14,27–29^. Thus, perturbation of the circadian system by non-genetic means in mice may be ideal for modeling the effects of circadian disruption on atherosclerosis in humans.

The goal of this study was to establish a model system to study the effects of light-induced circadian disruption on atherosclerosis. The ideal mouse model should have normal circadian rhythms and normal responsiveness to light and those rhythms should be susceptible to disruption by perturbation of the light-dark cycle. To this end, we first characterized circadian rhythms in *ApoE*^−/−^ mice. We chose to study *ApoE*^−/−^ mice because they spontaneously develop atherosclerosis^30^. Next, we sought to determine whether circadian disruption with constant light exposure increased atherosclerosis in *ApoE*^−/−^ mice.

## Results

### Circadian behavioral rhythms are similar in *ApoE*^+/+^ and *ApoE*^−/−^ mice

**Figure 1:**
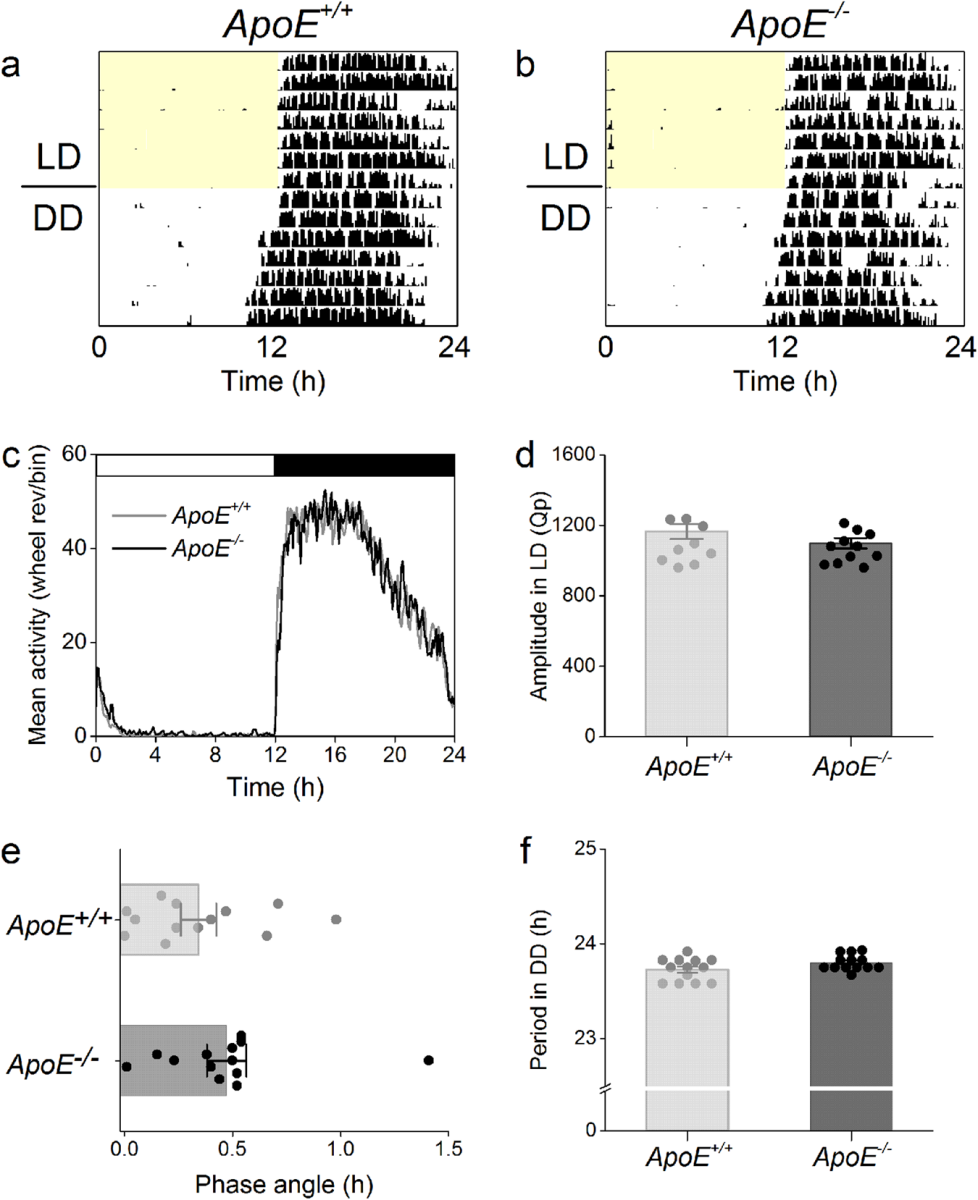
Circadian behavior rhythms in *ApoE*^−/−^ mice are indistinguishable from *ApoE*^+/+^ mice. Representative single-plotted actograms of wheel-running activity of *ApoE*^+/+^ (**a**) and *ApoE*^−/−^ (**b**) mice housed in 12 L:12D for 7 days (LD) and then released into constant darkness for 7 days (DD). Yellow shading shows lights on. Mean activity profiles (**c**) were generated from 7 days in 12 L:12D. The amplitudes in LD (**d**) were the peak Qp’s of the χ2 periodograms for 7 days in 12 L:12D. The phase angles of entrainment (**e**) were determined by drawing a regression line to activity onset for days 1–5 in constant darkness and then extending the line to the last day in 12 L:12D. A positive phase angle occurred when activity started after the time of lights off. The free-running period (**f**) was determined using an χ2 periodogram for days 1–7 in constant darkness. There were no significant differences between *ApoE*^+/+^ *and ApoE*^−/−^ mice. Data are mean ± SEM.

We first characterized daily and circadian wheel-running activity rhythms in C57BL/6 J *ApoE*^−/−^ mice. We found that *ApoE*^−/−^ mice had daily rhythms of wheel-running activity in 12 h light:12 h dark (12 L:12D) that were indistinguishable from *ApoE*^+/+^ mice (Fig. 1a,b; actograms of all individual mice shown in Figs. S1, S2). The amplitude of the wheel-running activity rhythm was not significantly different in *ApoE*^−/−^ and *ApoE*^+/+^ mice in 12 L:12D (Figs. 1c,d, Table S1, *t*-test *p* = 0.21). There was no significant difference between *ApoE*^−/−^ and *ApoE*^+/+^ mice in daily activity levels in 12 L:12D (Fig. S3a, Table S1, *t*-test *p* = 0.99).

We next released the mice into constant darkness to measure circadian behavior rhythms. We found that the phase angle of entrainment to the light-dark cycle was the same in *ApoE*^−/−^ and *ApoE*^+/+^ mice (Fig. 1e, Mann-Whitney *p* = 0.24). In constant darkness, the free-running period of wheel-running activity did not significantly differ between *ApoE*^+/+^ and *ApoE*^−/−^ mice (Fig. 1f, Table S1, *t*-test *p* = 0.08). The amplitude of the activity rhythm (Fig. S3d, *t*-test *p* = 0.03) and the daily activity level (Fig. S3b, *t*-test *p* = 0.05) were slightly decreased in *ApoE*^−/−^ mice in constant darkness (Table S1).

We next measured circadian behavior rhythms during the first 7 days in constant light (Fig. S4; actograms of all individual mice shown in Fig. S5). The free-running periods (Fig. S4c, Mann-Whitney *p* = 0.29) and amplitudes (Fig. S4d, *t*-test *p* = 0.46) of the wheel-running activity rhythms in constant light did not significantly differ between *ApoE*^+/+^ and *ApoE*^−/−^ mice (Table S1). The daily activity level in constant light was also similar between *ApoE*^+/+^ and *ApoE*^−/−^ mice (Fig. S3c, Table S1, *t*-test *p* = 0.92).

### Phase responses to light pulses are not altered in *ApoE*^−/−^ mice

**Figure 2:**
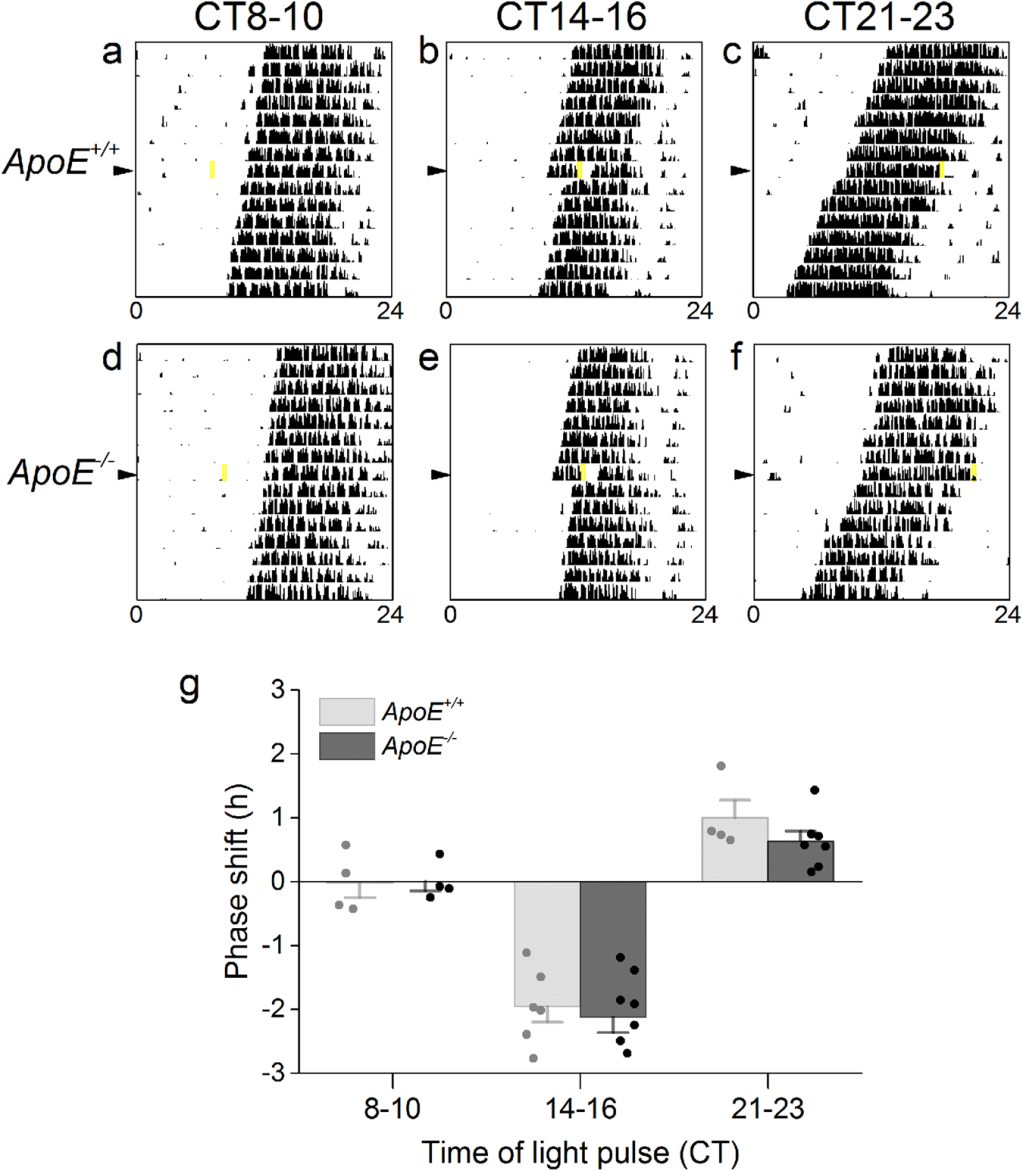
Phase responses to light pulses do not differ between *ApoE*^+/+^ and *ApoE*^−/−^ mice. Representative actograms of *ApoE*^+/+^ (**a–c**) and *ApoE*^−/−^ (**d–f**) mice administered a single 15-min light pulse (indicated by yellow box) at circadian time (CT) 8–10 (subjective day), 14–16 (early subjective night), or 21–23 (late subjective night) in constant darkness (x-axis: hours; y-axis: days). There were no significant differences between *ApoE*^+/+^ and *ApoE*^−/−^ mice (**g**). Data are mean ± SEM.

The phases (or timing) of circadian locomotor activity rhythms in C57BL/6 J mice shift in response to light exposure at certain times of day^31^. Light exposure during the early subjective night [at circadian time (CT) 14–16] delays the phase, while light during the late subjective night (CT21–23) advances the phase of the activity rhythm^31,32^. On the other hand, light during the subjective day (CT1–11) has no effect on the phase of the activity rhythm^31,32^. This light responsiveness is how the SCN entrains to the light-dark cycle and how the SCN adjusts to changes in the light-dark cycle (such as after travel to a new time zone). Thus, we next tested the phase responses to light pulses in *ApoE*^−/−^ mice. We housed *ApoE*^−/−^ and *ApoE*^+/+^ mice in constant darkness and then administered a single 15-min light pulse at either CT8–10, CT14–16, or CT21–23 (Fig. 2, light pulses for all individual mice are shown in Fig. S6, S7, S8). There were no significant differences in the magnitudes of the phase responses to light pulses between *ApoE*^−/−^ and *ApoE*^+/+^ mice at any phase (Table S1). Neither genotype had phase responses to light pulses at CT8–10 (*t*-test *p* = 0.94), and both genotypes phase-delayed to light pulses at CT14–16 (*t*-test *p* = 0.64) and phase-advanced to light pulses at CT21–23 (Mann-Whitney *p* = 0.16). These data demonstrate that *ApoE*^−/−^ mice have circadian light responsiveness that is indistinguishable from *ApoE*^+/+^ mice.

### Molecular timekeeping rhythms and circadian organization are not altered in *ApoE*^−/−^ mice

**Figure 3:**
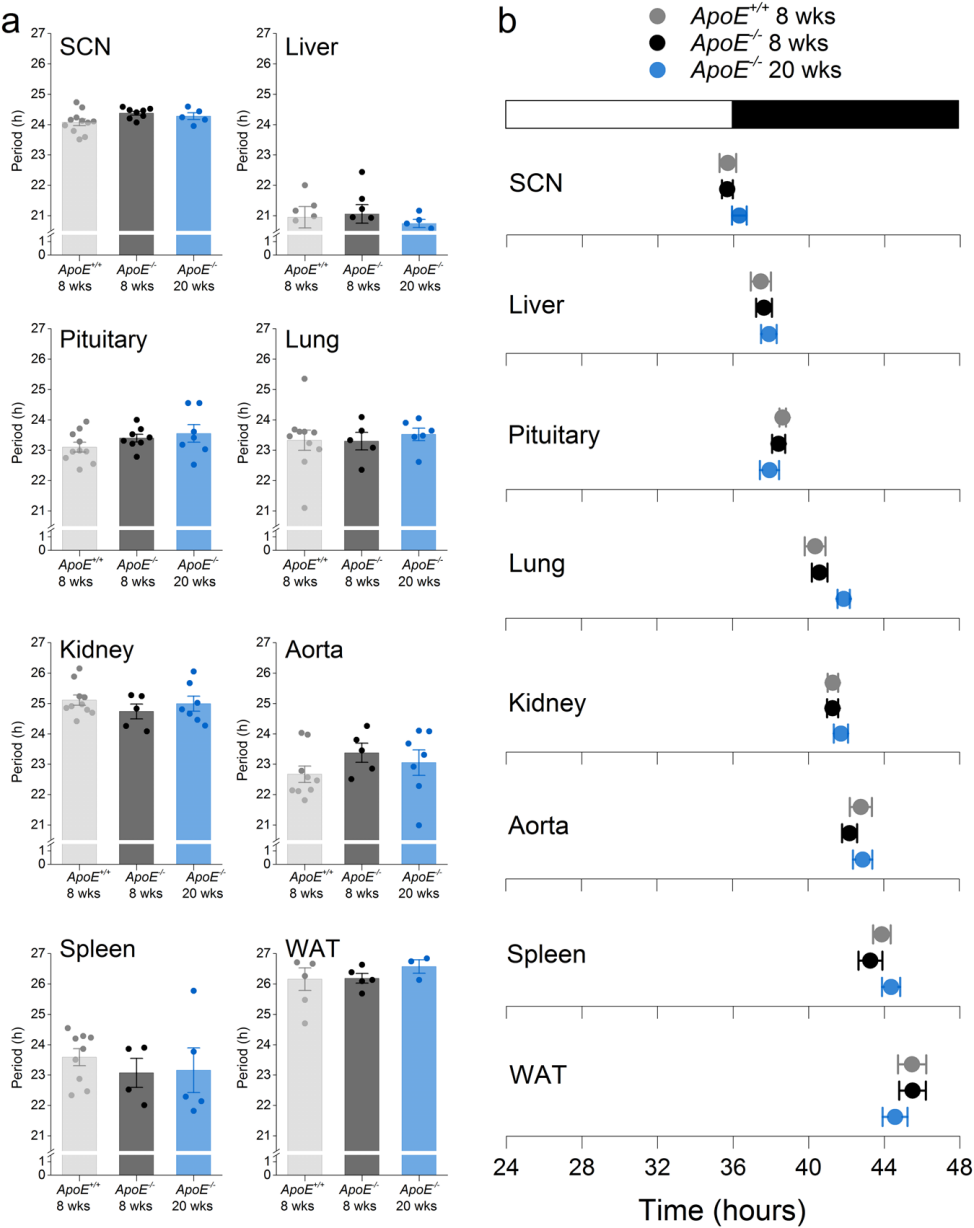
Molecular timekeeping rhythms in tissues are not altered in *ApoE*^−/−^ mice. Tissues were cultured from *ApoE*^+/+^ mice at 8 weeks old (light gray), *ApoE*^−/−^ mice at 8 weeks old (dark gray), and *ApoE*^−/−^ mice at 20 weeks old (blue) mice. The phases (mean ± SEM) were determined from the peaks of PER2::LUC expression during the interval between 12 and 36 h in culture and plotted relative to the last lights on. The white bar indicates lights on and black bar indicates lights off. SCN: suprachiasmatic nuclei; WAT: white adipose tissue. n = 5–10/tissue/group. There were no significant differences between *ApoE*^+/+^ and *ApoE*^−/−^ mice. Data are mean ± SEM.

We next measured the effect of *APOE* deficiency on the molecular timekeeping mechanism by measuring bioluminescence from tissues cultured from male and female *ApoE*^+/+^ and *ApoE*^−/−^ mice with the PERIOD2::LUCIFERASE reporter^33^ (Fig. 3, Fig. S9, Table S2). We analyzed tissues from *ApoE*^−/−^ mice at 8 and 20 weeks old because *ApoE*^−/−^ mice spontaneously develop atherosclerosis at ~12 weeks old, and we could therefore analyze tissue rhythms before and after development of atherosclerosis^34^. The periods of the PER2::LUC rhythms in SCN (*p* = 0.10), liver (*p* = 0.76), pituitary (*p* = 0.25), lung (*p* = 0.88), kidney (*p* = 0.51), aorta (*p* = 0.38), spleen (*p* = 0.68), and white adipose tissue (*p* = 0.66) did not differ significantly between 8-week old *ApoE*^+/+^ mice and *ApoE*^−/−^ mice at 8 and 20 weeks old (one-way ANOVA; Fig. 3a, Table S2). Likewise, we found that the phases of the SCN (*p* = 0.54), liver (*p* = 0.87), pituitary (*p* = 0.36), lung (*p* = 0.15), kidney (*p* = 0.59), aorta (*p* = 0.62), spleen (*p* = 0.41), and white adipose tissue (*p* = 0.73) were not significantly different between *ApoE*^+/+^ and *ApoE*^−/−^ mice at 8 and 20 weeks old (one-way ANOVA, Fig. 3b, Table S2). The amplitudes of the tissue PER2::LUC rhythms were also similar between 8-week old *ApoE*^+/+^ mice and *ApoE*^−/−^ mice at 8 and 20 weeks old (Table S2). These data demonstrate that the molecular timekeeping mechanism is not altered by *APOE* deficiency.

### Exposure to constant light exacerbates atherosclerosis in male *ApoE*^−/−^ mice

**Figure 4:**
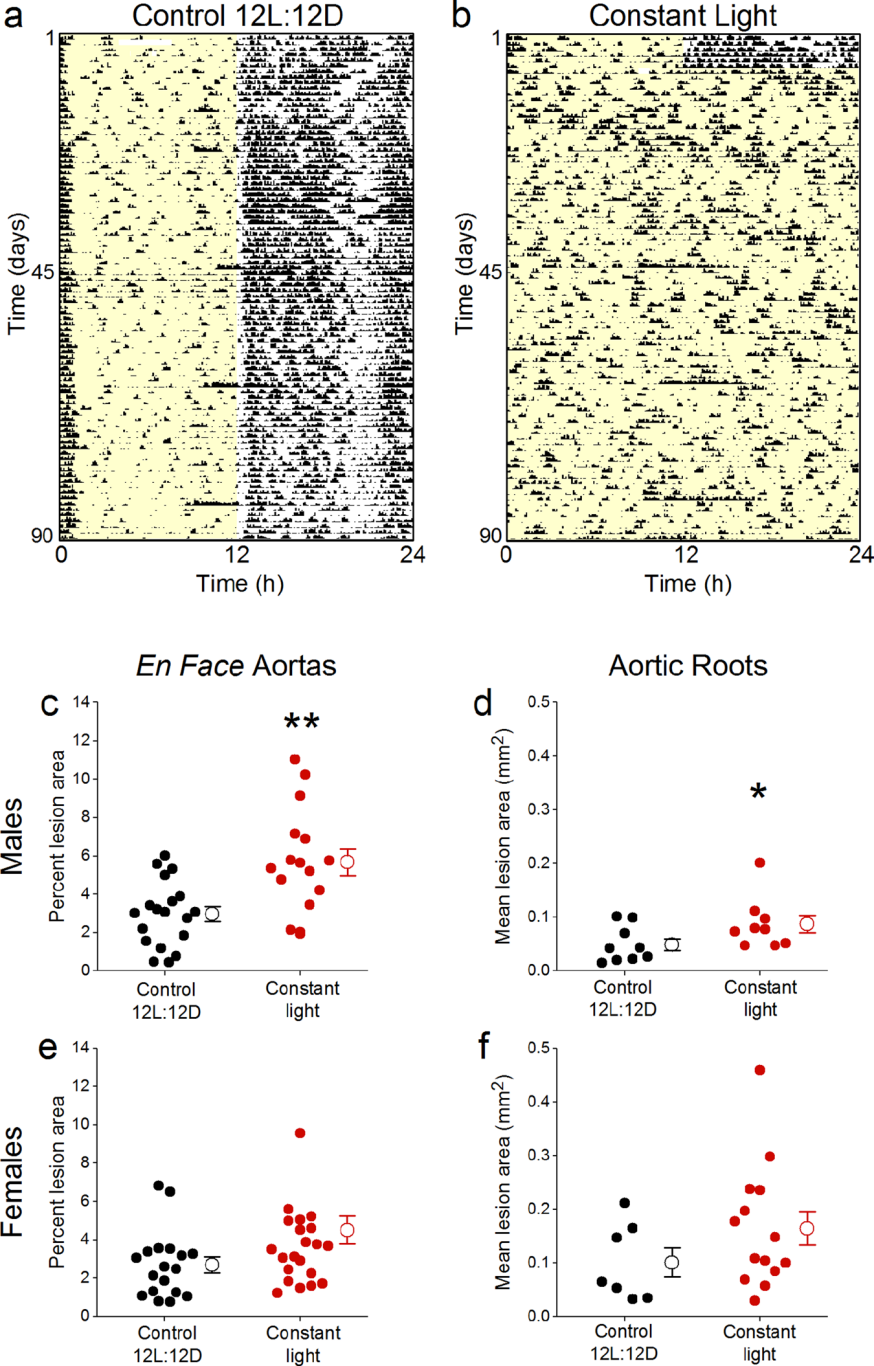
Constant light exposure exacerbates atherosclerosis in male *ApoE*^−/−^ mice. Representative single-plotted actograms of general locomotor activity of male *ApoE*^−/−^ mice housed in control 12 L:12D (**a**) or constant light (**b**) for 12 weeks. Yellow shading shows lights on. Percent atherosclerotic lesion area in *en face* aortas (**c,e**) and mean lesion area of aortic roots (**d,f**) of male (**c,d**) and female (**e,f**) *ApoE*^−/−^ mice housed in control 12 L:12D (black symbols) or constant light (red symbols) for 12 weeks. Data from individual mice are closed symbols and open symbols are mean ± SEM (**c–f**). *p < 0.05, **p = 0.001.

We next investigated the effects of chronic exposure to constant light on atherosclerosis (Fig. 4, Table S3). Similar to previous studies in wild-type mice, locomotor activity was arrhythmic or the rhythm was disrupted in *ApoE*^−/−^ mice housed in constant light (Fig. 4b, actograms of all individual mice shown in Fig. S10, S11, S12, S13; Fig. S14)^35^. Male (Fig. 4c, *t*-test *p* = 0.001), but not female (Fig. 4e, Mann-Whitney *p* = 0.08) *ApoE*^−/−^ mice housed in constant light had more atherosclerosis in the *en face* aorta compared to those in control 12 L:12D (Table S3). Likewise, atherosclerotic lesion area in the aortic root was increased in male (Fig. 4d, Mann-Whitney *p* = 0.04), but not in female (Fig. 4f, *t*-test *p* = 0.20), *ApoE*^−/−^ mice exposed to constant light (Table S3).

### Constant light exposure exacerbates dyslipidemia, but not inflammation in male *ApoE*^−/−^ mice

We next examined the potential mechanisms by which exposure to constant light could increase atherosclerosis in male *ApoE*^−/−^ mice. Male *ApoE*^−/−^ mice had similar body weights (*t*-test p = 0.13) and calorie consumption (*t*-test *p* = 0.52) in 12 L:12D and constant light, although the mice were less active in constant light (Mann-Whitney *p* = 0.008) (Table S4).

**Figure 5:**
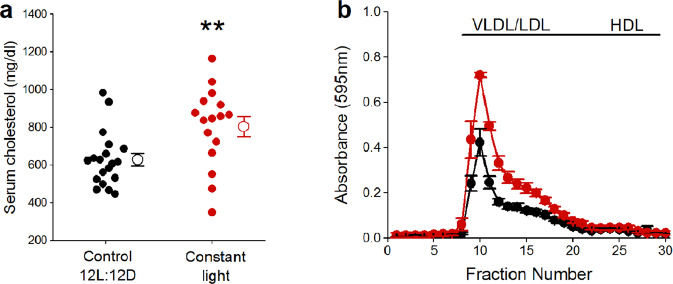
Constant light exposure increases VLDL/LDL-cholesterol concentrations in male *ApoE*^−/−^ mice. Total serum cholesterol concentrations (a: individual mice are closed symbols and open symbols are mean ± SEM) and distribution of cholesterol on lipoproteins (b: mean ± SEM, FPLC was performed on n = 4–5/group) of male *ApoE*^−/−^ mice housed in control 12 L:12D (black) or constant light (red) for 12 weeks. **p = 0.009.

Male *ApoE*^−/−^ mice housed in constant light had increased total serum cholesterol concentrations (Fig. 5a, Mann-Whitney *p* = 0.009), due to increased VLDL/LDL particles (Fig. 5b), compared to males in control 12 L:12D. There was no significant difference in total serum cholesterol concentrations (Fig. S15a, *t*-test *p* = 0.47) or in the distribution of cholesterol on lipoproteins (Fig. S15b) in female *ApoE*^−/−^ mice housed in control 12 L:12D compared to constant light (Table S3). Exposure to constant light did not significantly alter total serum triglycerides concentrations in male (Fig. S16a, Mann-Whitney *p* = 0.07) or female *ApoE*^−/−^ mice (Fig. S16b, *t*-test *p* = 0.40) (Table S3).

**Figure 6:**
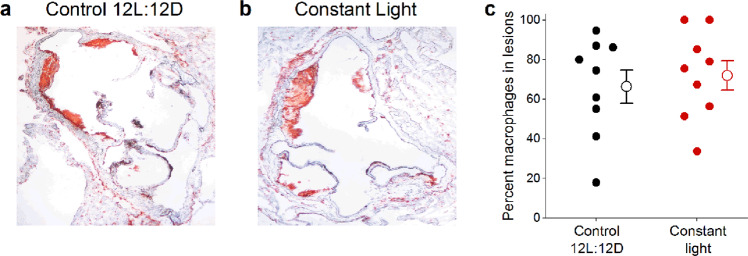
Constant light exposure does not increase macrophages in atherosclerotic lesions in aortic roots of male *ApoE*^−/−^ mice. Representative CD68 immunostaining of aortic roots (**a,b**) and percent of atherosclerotic lesions with CD68 staining (**c**) from male *ApoE*^−/−^ mice housed in control 12 L:12D (a, black symbols) or constant light (**b**, red symbols) for 12 weeks. Data from individual mice are closed symbols and open symbols are mean ± SEM (**c**). There was no significant difference between *ApoE*^+/+^ and *ApoE*^−/−^ male mice.

We next measured macrophages in the vascular wall of aortic roots as a marker of inflammation (Fig. 6). The percent macrophage content of the lesions in aortic roots was not altered by constant light exposure in male *ApoE*^−/−^ mice compared to mice in 12 L:12D (Fig. 6c, *t*-test *p* = 0.62, Table S3).

## Discussion

*ApoE*^−/−^ mice are a well-established rodent model for studying atherosclerosis^36^. Beginning at ~12 weeks of age, male and female *ApoE*^−/−^ mice spontaneously develop atherosclerotic lesions in the aorta, even when fed a low-fat (non-Western) diet^34^. It was critical to our experimental design to use a mouse model that develops atherosclerosis on low-fat diet. This is because diet-induced obesity increases atherosclerosis in *ApoE*^−/−^ mice and high-fat diet feeding disrupts daily rhythms in male wild-type mice^37,38^. Therefore, in this study, we sought to exclude the potential confounding effects of high-fat diet feeding on atherosclerosis and circadian rhythms in order to isolate the effects of constant light-induced circadian disruption. To this end, we fed *ApoE*^−/−^ mice a low-fat diet for the duration of the study. This protocol prevented hyperphagia and obesity in male *ApoE*^−/−^ mice. Thus, the exacerbation of atherosclerosis in male *ApoE*^−/−^ mice housed in constant light occurred independently of systemic metabolic dysfunction caused by obesity.

The first goal of this study was to determine if *ApoE*^−/−^ mice are an appropriate model for studying the effects of light-induced circadian disruption on atherosclerosis. An ideal model should have no inherent circadian rhythm abnormalities. One previous study found that *ApoE*^−/−^ mice had unstable activity rhythms in constant darkness and impaired circadian responsiveness to light^39^. Therefore, we first comprehensively characterized circadian rhythms of behavior and light responsiveness, and molecular circadian rhythms in tissues in *ApoE*^−/−^ mice. We found that *ApoE*^−/−^ mice were indistinguishable from *ApoE*^+/+^ mice in every parameter measured, with the exception of slight significant reductions in total activity and amplitude in *ApoE*^−/−^ mice in constant darkness. Endogenous (free-running) and entrained rhythms of wheel-running activity, as well as responsiveness to light pulses at different times of day were the same in *ApoE*^−/−^ and *ApoE*^+/+^ mice. Moreover, the molecular timekeeping rhythms in the SCN and peripheral tissues, as well as the phase relationship between these body clocks, were the same in *ApoE*^−/−^ and *ApoE*^+/+^ mice. We postulate that our results differ from the previous study because the *ApoE*^−/−^ mice used in the prior study may have been on a mixed genetic background while the *ApoE*^−/−^ mice in our study were backcrossed 10 times into a C57BL/6 J background. Genetic background markedly affects circadian activity rhythms in mice and the C57BL/6 J strain has stable, high-amplitude behavior rhythms^40^. In sum, we found that *ApoE*^−/−^ mice have no circadian abnormalities and thus are an ideal model for studying the effects of light-induced circadian disruption on atherosclerosis.

The second goal of this study was to investigate the effects of constant light exposure, which chronically disrupts circadian rhythms, on atherosclerosis. Most previous studies investigated the effects of circadian disruption on atherosclerosis using circadian gene mutant mice^15–19^. To our knowledge, only two studies have examined the effects of light-induced circadian disruption on atherosclerosis in mice. One study inferred that a weekly inversion of the light-dark cycle accelerated atherosclerosis development in *ApoE*^−/−^ mice, but lacked quantitative data^41^. A second study recently showed that weekly inversion of the light-dark cycle increased severe atherosclerotic lesions in female APOE*3-Leiden.CETP mice due to increased macrophage content and inflammation, oxidative stress, and chemoattraction markers in the vascular walls^42^. However, the data from the present study demonstrate that the increase in atherosclerosis in male *ApoE*^−/−^ mice exposed to constant light was driven by increased VLDL/LDL cholesterol rather than increased macrophage content. Additionally, the mice in the previous study were fed a Western diet (high fat and high cholesterol), which independently causes metabolic dysfunction, obesity, and disrupts circadian rhythms^27,43–48^. Therefore, our study is unique in that we found that disordered light exposure exacerbates atherosclerosis even when mice are fed low-fat diet. An additional strength of our study is that we studied both male and female *ApoE*^−/−^ mice, while only females could be studied in the previous study because male APOE*3 Leiden.CETP mice do not develop atherosclerosis on a cholesterol-rich diet.

In mice, constant light exposure increases the period of activity rhythms and, chronically, can cause arrhythmicity^49^. At the tissue level, constant light desynchronizes cellular oscillators in the SCN, causing the overall rhythm of the SCN to be low-amplitude or absent^35^. Since the SCN is the master circadian clock that coordinates physiological and behavioral rhythms throughout the body, the effect of constant light exposure on the SCN results in whole-body disruption of circadian rhythms^35,50^. Previous studies showed that constant light exposure increased body weight, disrupted insulin sensitivity, and decreased triglyceride-derived fatty acids and glucose uptake by brown adipose tissue^51–53^. However, no study has examined the effects of constant light exposure on atherosclerosis. In the current study, we found that exposure to constant light increased cholesterol on atherogenic lipoproteins and atherosclerotic lesion area in male *ApoE*^−/−^ mice. According to the lipid hypothesis, chronic elevated levels of cholesterol in the blood causes atherosclerosis^54–56^. In female *ApoE*^−/−^ mice, constant light exposure did not increase total or VLDL/LDL-cholesterol concentrations nor atherosclerotic lesion area. The mechanisms underlying the sex difference in response of lipids to light-induced circadian disruption are unknown but could be due to differences in circulating sex hormones.

In sum, we found that *ApoE*^−/−^ mice had circadian rhythms that were indistinguishable from *ApoE*^+/+^ mice. In addition, circadian disruption with constant light exposure increased atherosclerosis in male, but not female *ApoE*^−/−^ mice. Together these data establish male *ApoE*^−/−^ mice as an appropriate model for studying the effects of light-induced disruption of circadian rhythms on atherosclerosis.

## Methods

### Animals

C57BL/6 J *ApoE*^−/−^ (N10) mice were purchased from The Jackson Laboratory (stock # 002052) and bred with wild-type C57BL/6 J mice (from The Jackson Laboratory) to generate C57BL/6 J heterozygous *ApoE*^*+/-*^ mice. Heterozygous C57BL/6 J *ApoE*^*+/-*^ males and females were bred to generate *ApoE*^+/+^ and *ApoE*^−/−^ mice (N11-N12) for experiments. For bioluminescence experiments, *ApoE*^*+/-*^ mice that were heterozygous for PERIOD2::LUCIFERASE^33^ (originally obtained from Dr. Joseph Takahashi and then backcrossed for 25 generations with C57BL/6 J mice from The Jackson Laboratory) were crossed with *ApoE*^*+/-*^ mice to generate *ApoE*^+/+^ and *ApoE*^−/−^ mice that were heterozygous for PER2::LUC for experiments. Breeders and weanlings were housed in 12 L:12D and fed standard rodent laboratory diet (Teklad 2918) and water *ad libitum*. At 3 weeks old, offspring were weaned and group-housed with same-sex siblings. Genotyping for *ApoE*^−/−^ was performed according to the protocol on The Jackson Laboratory website. Genotyping for PER2::LUC was determined by measuring bioluminescence from tail snips. For all experiments, mice were singly-housed in cages (33 cm × 17 cm × 14 cm) with running wheels (diameter: 11 cm) and fed low-fat diet (Research Diets D12450K, 10% kcal fat) and water *ad libitum*. The running wheels were either unlocked (could rotate) or locked (could not rotate), as indicated for each specific experiment below. All procedures were conducted in accordance with animal protocol 2015–2211 approved by the University of Kentucky Institutional Animal Care and Use Committee.

### Characterization of circadian behavior

At 7 to 8 weeks old, male and female *ApoE*^−/−^ and *ApoE*^+/+^ mice were housed singly in cages with unlocked running wheels. The cages were placed in light-tight boxes in 12 L:12D with white LED lights (intensity 250 to 350 lux). Wheel revolutions were recorded every minute using the ClockLab system (Actimetrics, Inc, Wilmette, IL). Mice were housed in 12 L:12D for 7 days and then in constant darkness for 7 days. Data were analyzed with ClockLab analysis software (Actimetrics). Mean activity profiles of wheel-running activity (5-min bins) were compiled for7 days in 12 L:12D. The amplitude (Q_p_) of the wheel-running rhythm was the peak value of the χ^2^ periodogram for 7 days in 12 L:12D or 7 days in constant darkness or 7 days in constant light. Mean daily activity was determined for 7 days in 12 L:12D or 7 days in constant darkness or 7 days in constant light. The phase angle of entrainment was determined by fitting a linear regression line to 5 days in constant darkness, and then extending it back to the last day in 12 L:12D. The free-running period of wheel-running activity in constant darkness was determined by χ^2^ periodogram with alpha set to 0.001 for days 1–7 in constant darkness. After 18 days in constant darkness with weekly light pulses (see below), we returned the mice to 12 L:12D for 20 days and then released them into constant light for 21 days. The free-running period of wheel-running activity in constant light was determined by χ^2^ periodogram for days 1–7 in constant light. Wheel-running activity data are shown in actograms in 6-min bins in the normalized format (ClockLab).

### Circadian phase responses to light pulses

Male and female *ApoE*^+/+^ and *ApoE*^−/−^ mice (12–18 weeks old at time of light pulse) were single housed in cages with *ad libitum* access to unlocked running wheels. The cages were housed in light-tight boxes in 12 L:12D with white LED lights (intensity 250 to 350 lux). Mice were then released into constant darkness for 7 days. The onset of activity on the day of the light pulse, which was designated as CT12, was determined by linear regression using ClockLab Analysis. A single light pulse (15 min, 150 lux white LED) was administered at CT8–10 (subjective day), or CT14–16 (early subjective night), or CT21–23 (late subjective night). The mice then free-ran for 7 days after the light pulse. The magnitudes of the phase shifts were determined by measuring the time between a line fit to the onset of activity the 7 days before the light pulse and a line fit to the onset of activity the 7 days after the light pulse using ClockLab Analysis software. Some mice were administered more than 1 light pulse, every 3 weeks, and the cages were changed in the week between pulses. Each mouse received no more than 3 light pulses and an individual mouse never received a light pulse at the same CT.

### Bioluminescence tissue rhythms

Male and female *ApoE*^−/−^ and *ApoE*^+/+^ mice (7 weeks old) that were heterozygous for PER2::LUC were housed singly in cages with locked wheels (wheels were present but could not rotate) in 12 L:12D. At 8 or 20 weeks old, mice were euthanized by cervical dislocation without anesthesia and aorta, kidney, lung, liver, pituitary, spleen, SCN, and white adipose tissue explants were dissected and cultured as described previously^57^. Bioluminescence was measured every 10 min with the 32-channel LumiCycle apparatus (Actimetrics Inc.). Data were smoothed by 30-min adjacent averaging and detrended using LumiCycle Analysis software (Actimetrics Inc.). The amplitude (goodness of fit ≥90%) was determined from the cycle that occurred between 12–36 hours in culture with LumiCycle Analysis software (Actimetrics). The data were exported to ClockLab analysis software to analyze period and phase. The period was determined from a regression line fit to the acrophase of 3–5 cycles. The phase was the acrophase of the peak of bioluminescence that occurred between 12–36 hours in culture.

### Effects of constant light exposure on atherosclerosis, lipids, and inflammation

At 7 weeks old, male and female *ApoE*^−/−^ mice were single-housed in cages with locked running wheels in light-tight boxes in 12 L:12D. General locomotor activity was continuously recorded with passive infrared sensors and collected in one-minute intervals using the ClockLab acquisition system (Actimetrics Inc.). At 8 weeks old, mice were randomized to either remain in 12 L:12D or to be housed in constant light for 12 weeks. Body and food weights were measured weekly between ZT9-ZT12, where ZT0 is lights on and ZT12 is lights off, in 12 L:12D, or the corresponding local time for mice in constant light. χ^2^ periodograms were used to determine whether general locomotor activity was rhythmic in the final 28 days in constant light using ClockLab Analysis software. Activity was rhythmic if a dominant peak exceeded alpha set at *p* = 0.001 (period range 20–36 h).

At 20 weeks of age, mice were anesthetized with inhaled isoflurane between ZT6–9 (or the corresponding local time for mice in constant light) until unresponsive to toe pinches, then euthanized by cervical dislocation. Blood was collected by cardiac puncture and serum was separated by centrifugation and stored at −80 °C. The aortas were perfused with NaCl (0.9% wt/vol) via left ventricular puncture. Aortas were dissected from the root to the iliac bifurcation as described previously^58^. The heart was removed from the aorta and stored at −80 °C. Mice with gross organ abnormalities were excluded from the study (2 mice from the female constant light group were excluded for enlarged kidneys). The aortas were stored in 10% neutrally-buffered formalin for 24-hours at room temperature and then transferred to 0.9% NaCl solution and stored at room temperature.

To measure atherosclerotic lesion area in *en face* aortas, peri-aorta adipose tissue was removed and the aorta was cut longitudinally and photographed using Image-Pro 7.0 software. The aortic arch was defined as the ascending arch to 3 mm distal to the root of the left subclavian artery. Atherosclerotic lesion area of the aortic arch was measured using Image-Pro 7.0 software by one researcher not blinded to the experimental treatments and analyzed by a second researcher who was blinded to the experimental groups.

Atherosclerotic lesion area in the aortic root was measured as described previously^58^. Briefly, 9 serial tissue sections (10 µm) from the origin of the aortic valves to the ascending aorta, were stained with oil red O. Serial sections were distributed among eight consecutive slides resulting in ~80 µm intervals for each slide. Lesions were quantified from the internal elastic lamina to the luminal edge using Image-Pro 7.0 software by 2 researchers as described above.

### Lipid analysis

Total serum cholesterol (Cholesterol E Enzymatic Kit, Wako Pure Chemical Industries kits Mountain View, CA) and triglycerides concentrations (L-Type Triglyceride M Enzyme Color A and Color B, Wako Pure Chemical Industries kits Mountain View, CA) were analyzed. Lipoprotein cholesterol and triglyceride distributions were analyzed from individual serum samples (50 µl) fractioned by fast protein liquid chromatography (FPLC) (Leica CM1860). Fractions were collected and cholesterol and triglyceride concentrations were determined with the Wako kits described above. For each group, 4–5 samples around the mean total serum cholesterol concentration were analyzed.

### Macrophage quantification

Immunohistochemistry for macrophages was performed on frozen serial sections (adjacent to those stained with Oil Red O) of the ascending aorta using the MicroProbe system as described previously using rat anti-mouse CD68 (Bio-Rad, Cat# MCA1957), Rat IgG2b (BD PharMingen, Cat# 559478), and biotinylated rabbit anti-rat IgG (Vector, Cat# BA-4001) antibodies^59^. Immunoreactivity was visualized with red chromogen 3-amino-9-ethylcarbazole (AEC) (Vector). Macrophage content was quantified in the first serial section of the aortic root from the internal elastic lamina to the luminal edge using Image-Pro 7.0 software by 2 researchers as described above. The area of CD68 staining was expressed as a percentage of atherosclerotic lesion area.

### Statistical analyses

A priori power analyses were performed for circadian behavior parameters and *en face* atherosclerotic lesion area using alpha = 0.05, power = 0.80, and effect size of 1 to 1.5 using G*Power (Heinrich Heine Universität Düsseldorf) (Table S5). Circadian behavior parameters (amplitude, daily activity, phase angle of entrainment, period) and phase shifts in reponse to light pulses at each CT were compared between *ApoE*^+/+^ and *ApoE*^−/−^ mice using two-tailed Student’s *t*-tests, unless the data were not normally distributed or had unequal variance, in which case the Mann-Whitney test was used. One-way ANOVAs were used to compare the periods, phases, and amplitudes of the bioluminescence rhythms of each tissue among groups. Atherosclerosis lesion area in the *en face* aorta and aortic roots, total serum cholesterol and triglyceride concentrations, cumulative food intake, cumulative locomotor activity, and the percentage of lesions with macrophages in *ApoE*^−/−^ mice of the same sex were compared between 12 L:12D and constant light conditions using two-tailed Student’s *t*-tests, unless the data were not normally distributed or had unequal variance, in which case the Mann-Whitney test was used. Statistical tests were performed with OriginPro 2016 (Northampton, MA). Data are presented as the mean ± SEM. Significance was ascribed at *p* < 0.05.

## Supplementary information

Supplementary Information.

## Data Availability

All data generated or analyzed during this study are included in this published article and its Supplementary Information files.
