# Supplementary material for: Circadian disruption with constant light exposure exacerbates atherosclerosis in male ApolipoproteinE-deficient mice
Source: Sci Rep. 2020 Jun 18;10:9920. doi: 10.1038/s41598-020-66834-9 (PMC7303111; doi:10.1038/s41598-020-66834-9)
Supplement: Supplementary file 1 — Supplementary Information. [file 41598_2020_66834_MOESM1_ESM.pdf]

Supplementary Information

Circadian disruption with constant light exposure exacerbates atherosclerosis in male *ApolipoproteinE*-deficient mice

Jeffrey M. Chalfant, Deborah A. Howatt, Lisa R. Tannock, Alan Daugherty,
and Julie S. Pendergast

Supplementary Figures

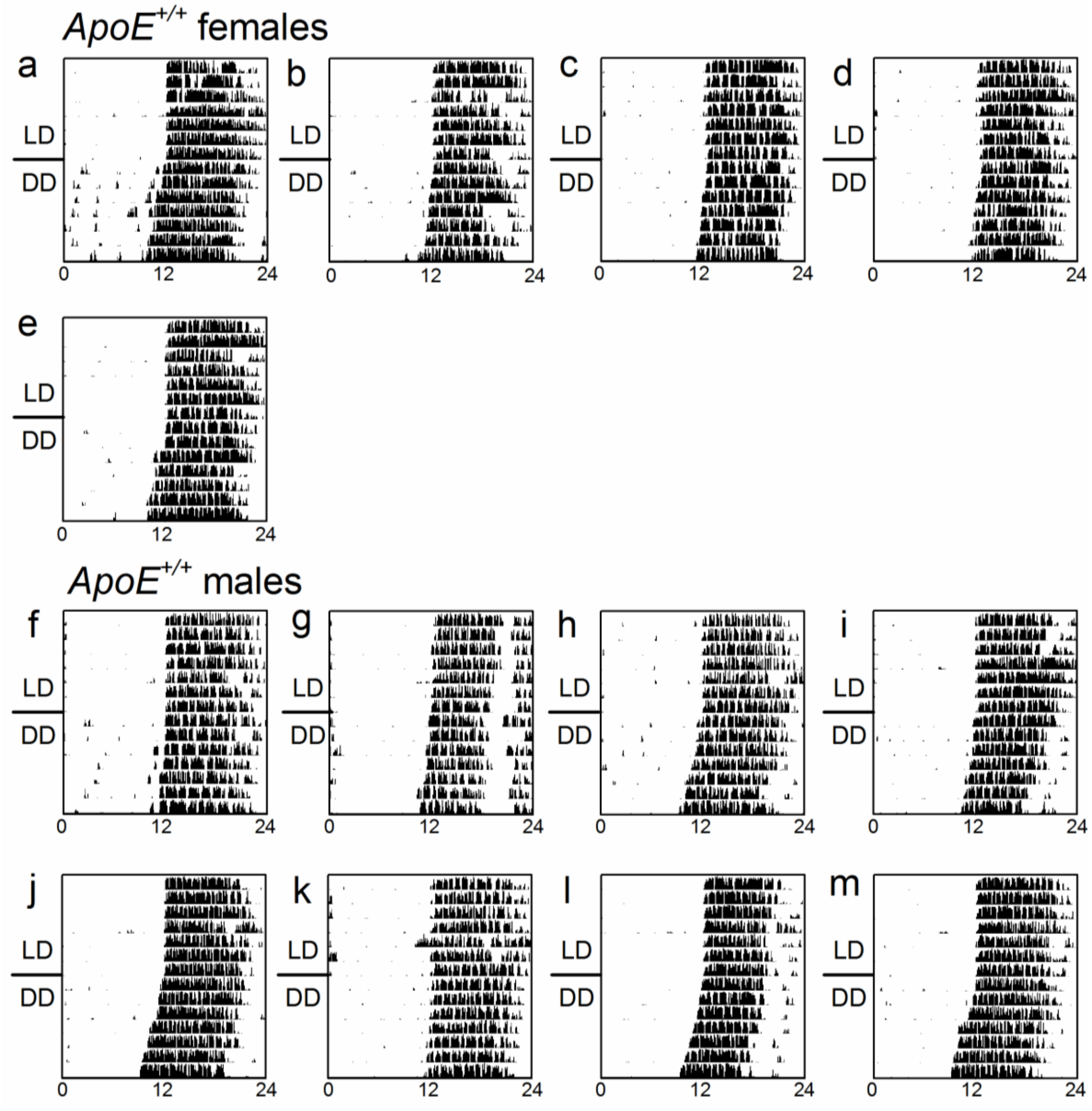

Figure S1. Wheel-running activity of *ApoE*$^{+/+}$ mice in 12L:12D and constant darkness. Single-plotted actograms of wheel-running activity of individual female (a-e) and male (f-m) *ApoE*$^{+/+}$ mice. Mice were housed in 12L:12D (LD, 0-12h is lights on and 12-24h is lights off) for 7 days and then released into constant darkness (DD) for 7 days. x-axis: hours; y-axis: days.

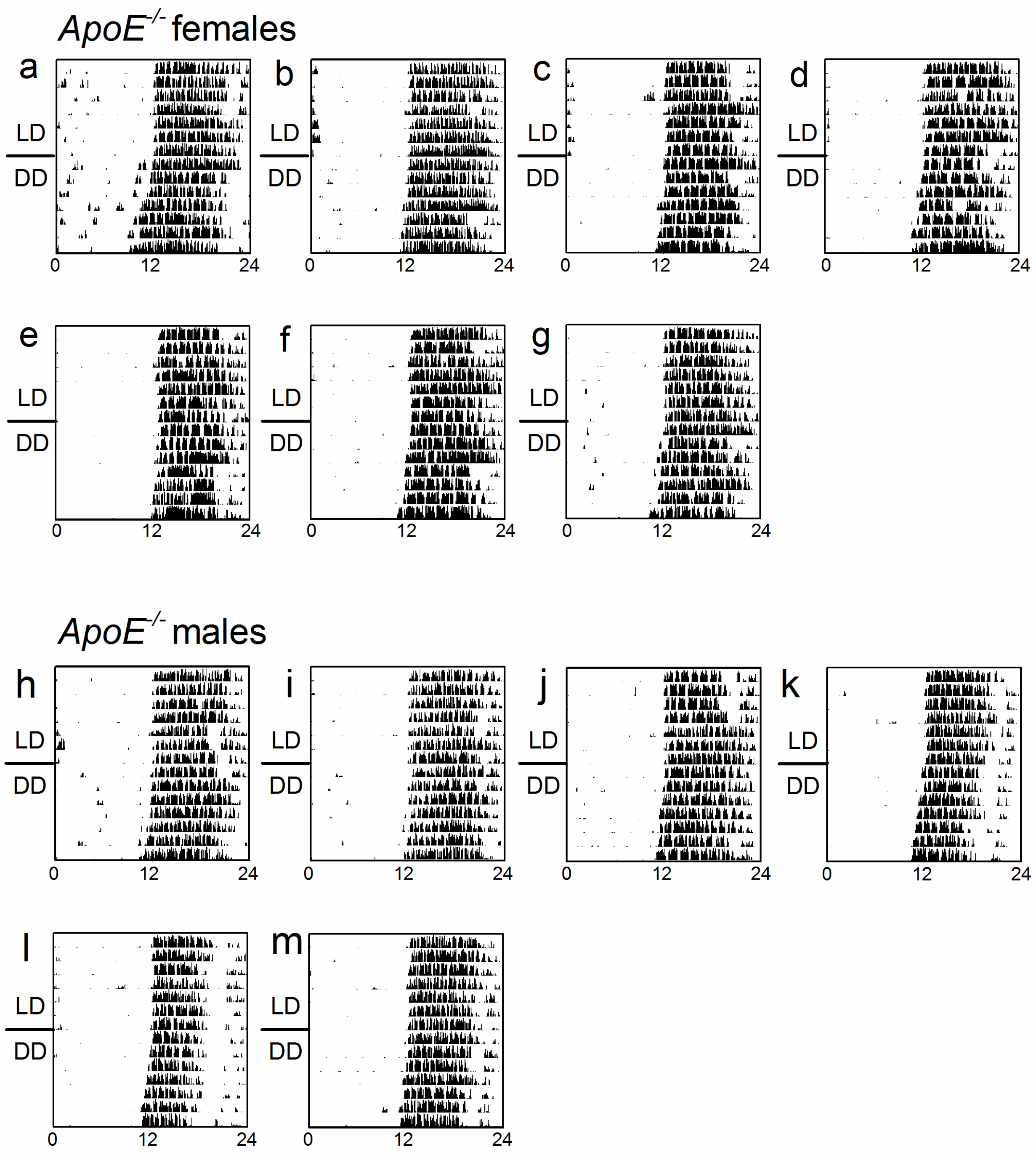

Figure S2. Wheel-running activity of *ApoE$^{-/-}$* mice in 12L:12D and constant darkness. Single-plotted actograms of wheel-running activity of individual female (a-g) and male (h-m) *ApoE$^{-/-}$* mice. Mice were housed in 12L:12D (LD, 0-12h is lights on and 12-24h is lights off) for 7 days and then released into constant darkness (DD) for 7 days. x-axis: hours; y-axis: days.

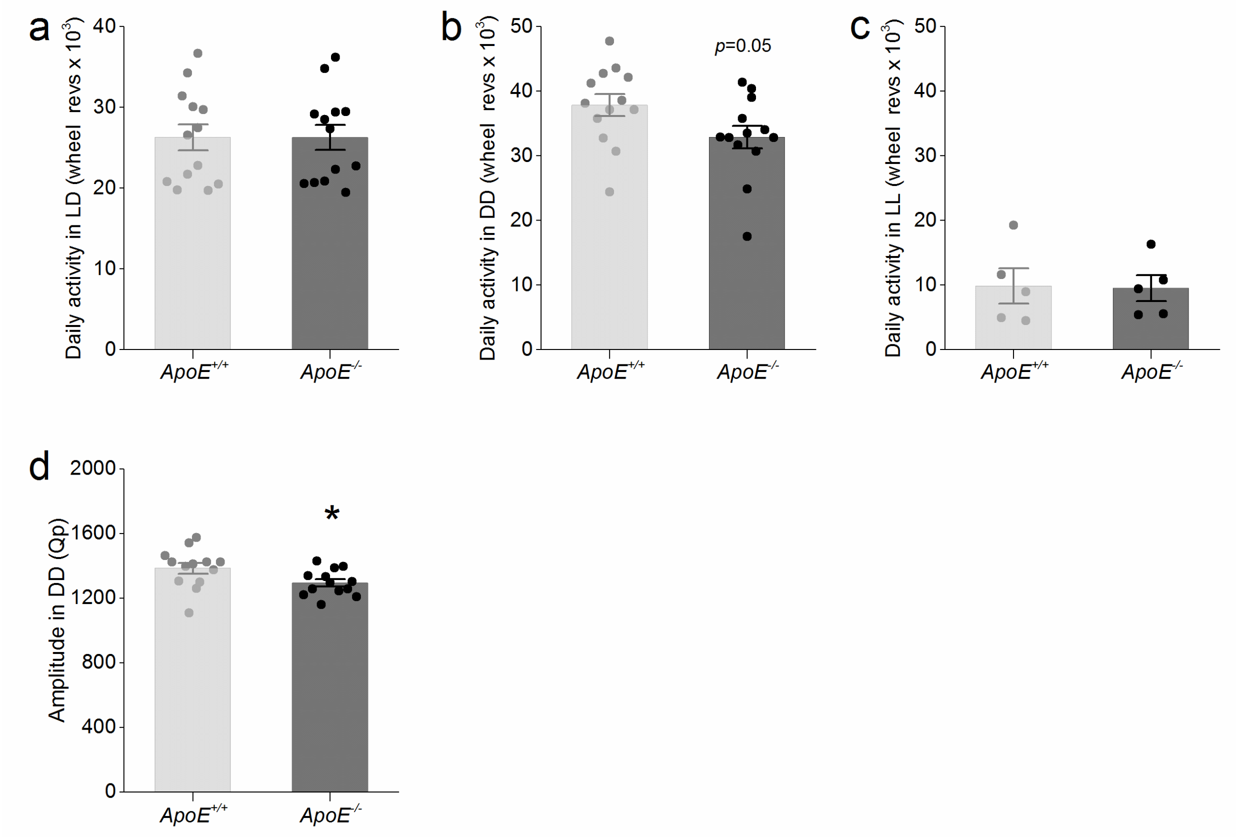

Figure S3. Daily activity levels and amplitudes of the locomotor activity rhythms in *ApoE*$^{+/+}$ and *ApoE*$^{-/-}$ mice. Daily activity (wheel revolutions x 10^3) was averaged over 7 days in 12L:12D (a, LD), constant darkness (b; DD) or constant light (LL). The amplitudes (Qp) of the wheel-running activity rhythms in DD were analyzed for days 1-7 in DD (d). *$p<0.05$

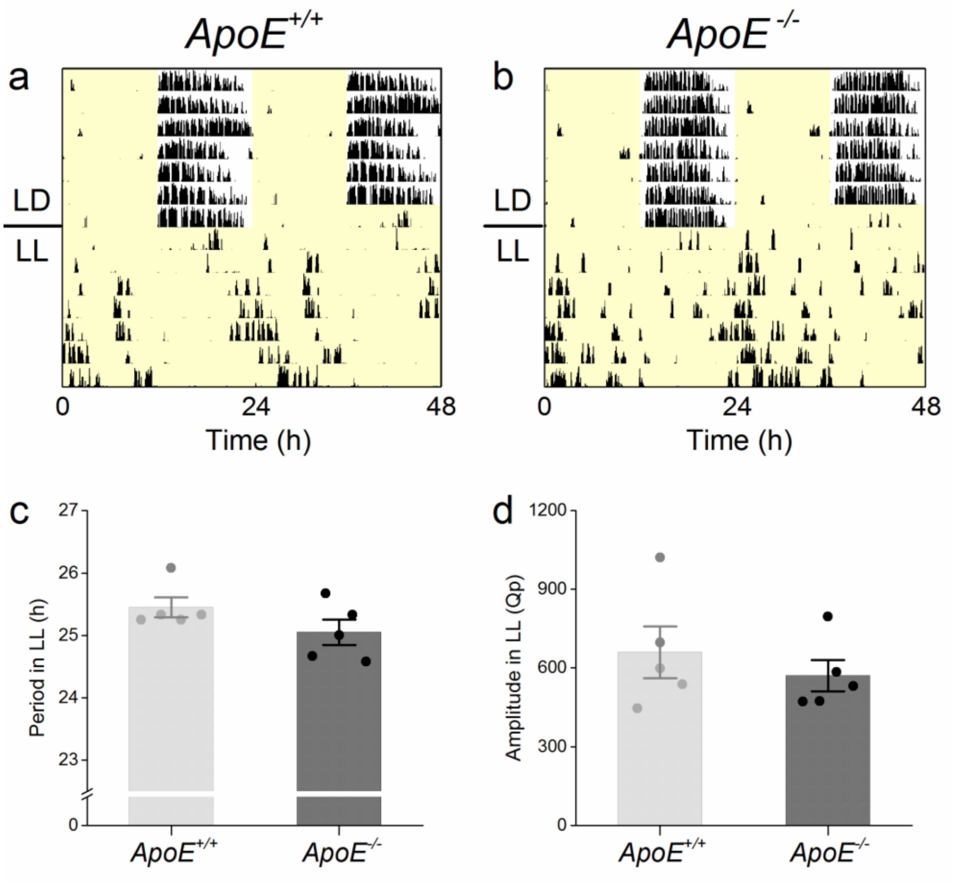

Figure S4. Circadian behavior rhythms in constant light in *ApoE*$^{-/-}$ mice are indistinguishable from *ApoE*$^{+/+}$ mice. Representative double-plotted actograms of wheel-running activity of *ApoE*$^{+/+}$ (a) and *ApoE*$^{-/-}$ (b) mice housed in 12L:12D for 7 days (LD) and then released into constant light for 7 days (LL). Yellow shading shows lights on. The free-running periods (c) and amplitudes (d) were determined using $\chi 2$ periodograms for days 1-7 in constant light. There were no significant differences between *ApoE*$^{+/+}$ and *ApoE*$^{-/-}$ mice. Data are mean ± SEM.

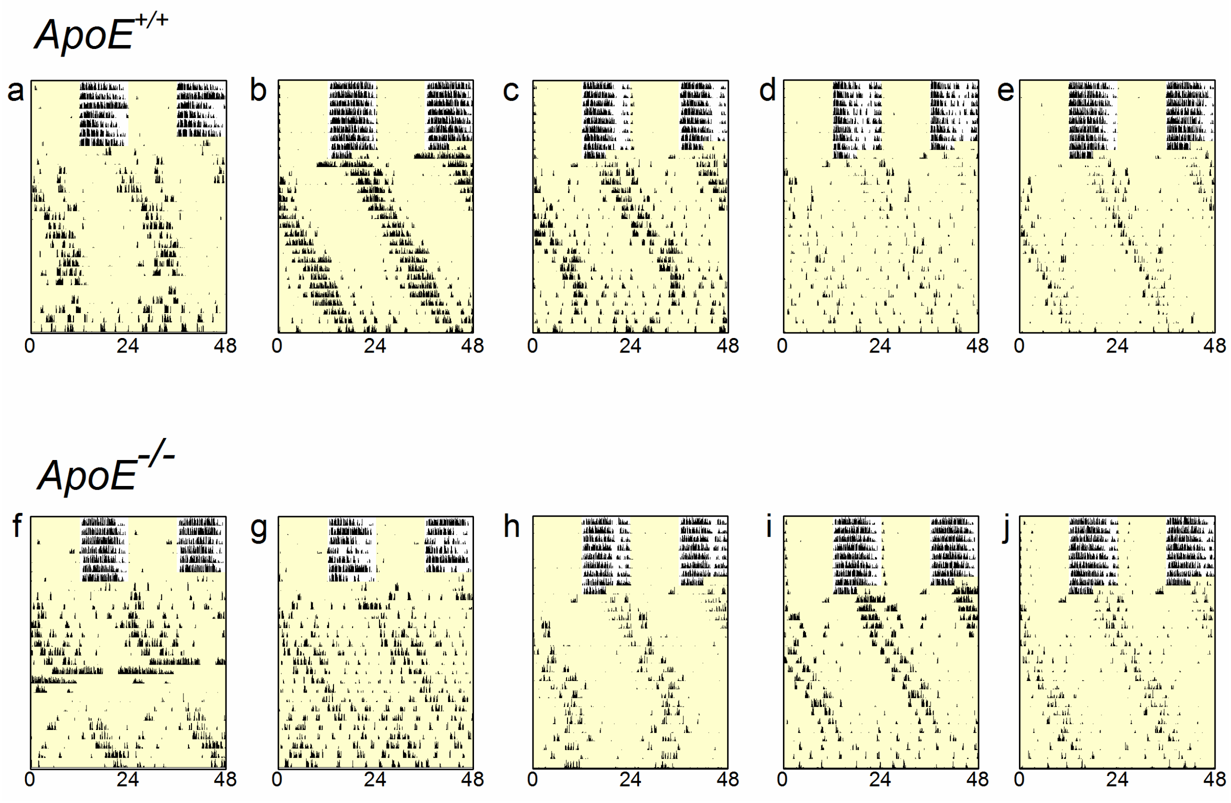

Figure S5. Wheel-running activity of *ApoE*$^{+/+}$ and *ApoE*$^{-/-}$ mice in constant light. Double-plotted actograms of wheel-running activity of *ApoE*$^{+/+}$ (a-e) and *ApoE*$^{-/-}$ (f-j) mice. Mice were housed in 12L:12D and then released into constant light for 21 days. Yellow shading shows lights on. x-axis: hours; y-axis: days. Females are shown in a, f, and g. Males are shown in b-e and h-j.

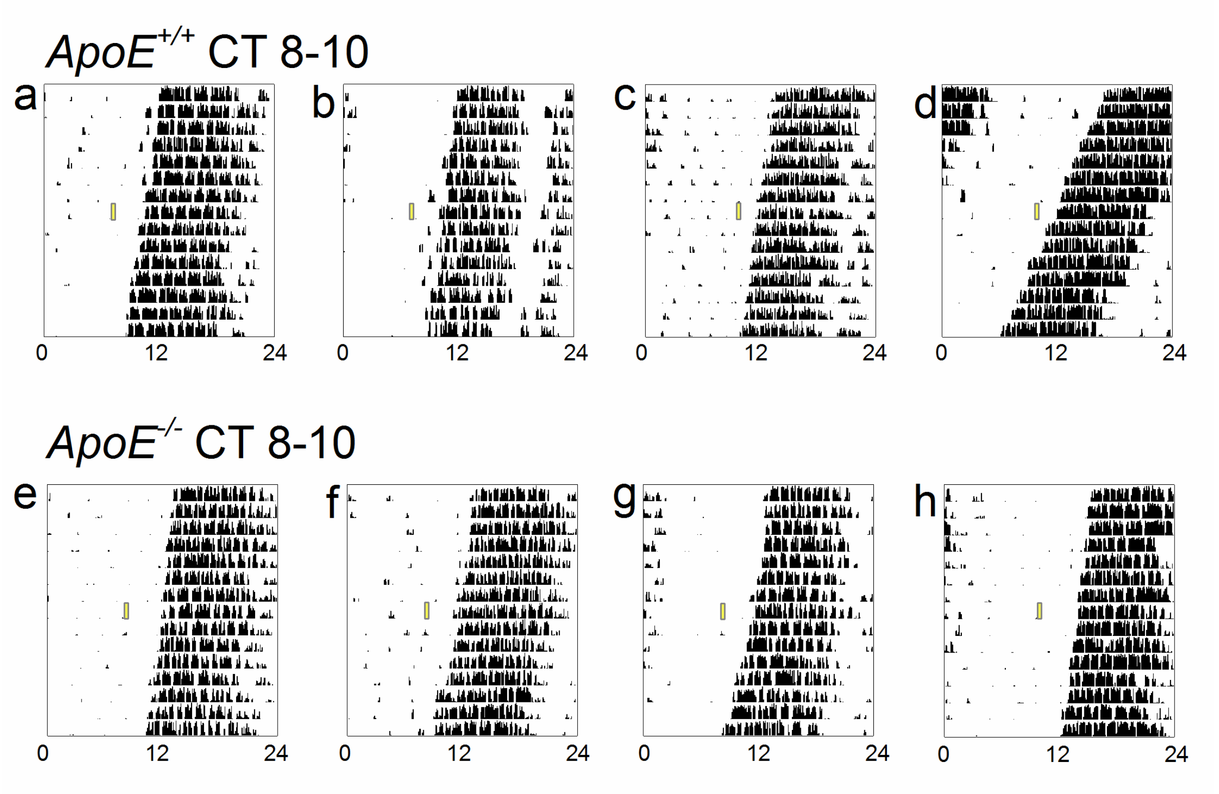

Figure S6. Phase responses to light pulses at CT 8-10 in *ApoE*$^{+/+}$ and *ApoE*$^{-/-}$ mice. Single-plotted actograms of *ApoE*$^{+/+}$ (a-d) and *ApoE*$^{-/-}$ (e-h) mice administered a single 15-min light pulse (yellow bar) at circadian time (CT) 8-10 (subjective day) in constant darkness (x-axis: hours; y-axis: days). Males are shown in a, b and e-g. Females are shown in c, d, and h.

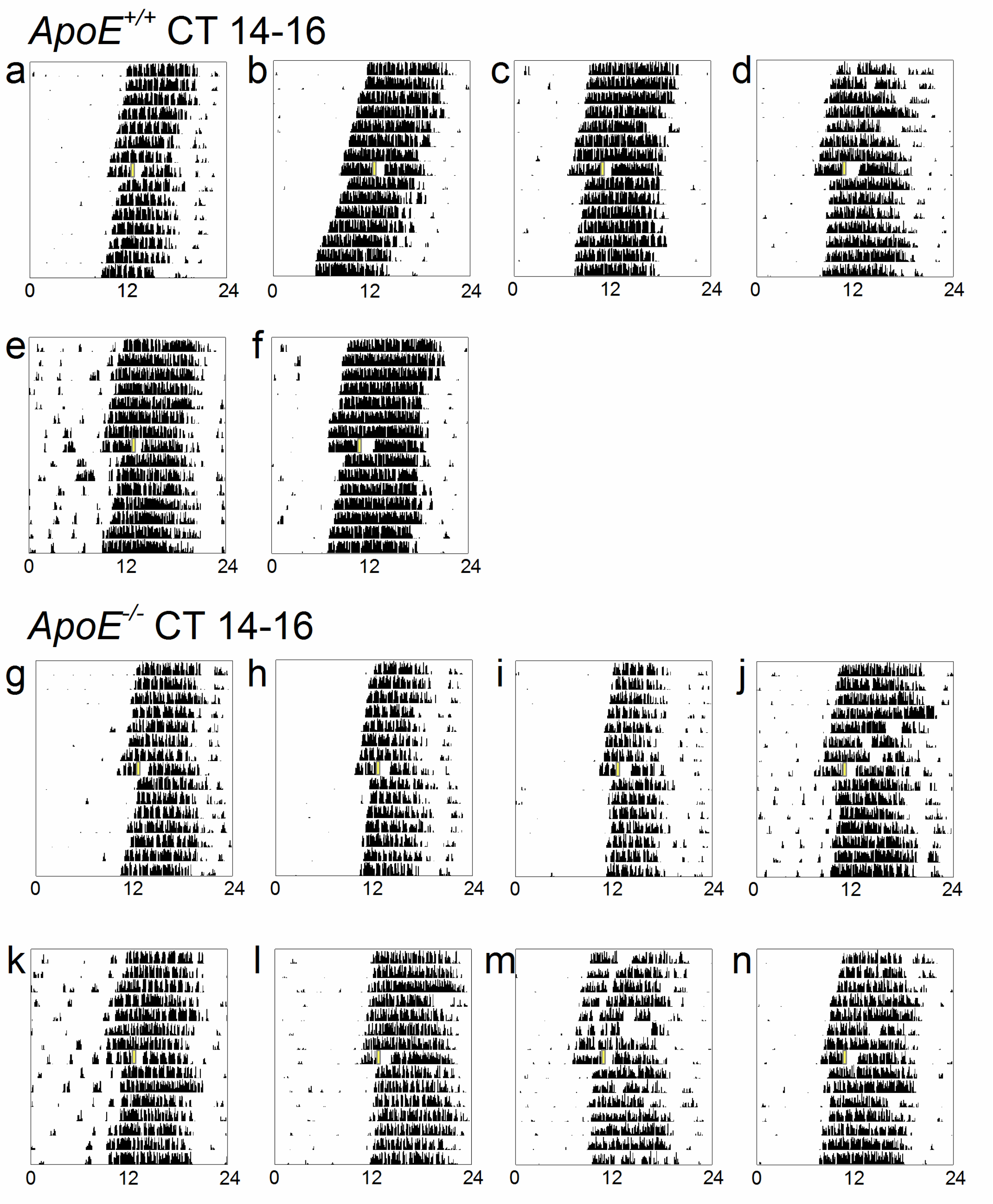

Figure S7. Phase responses to light pulses at CT 14-16 in *ApoE*$^{+/+}$ and *ApoE*$^{-/-}$ mice. Single-plotted actograms of *ApoE*$^{+/+}$ (a-f) and *ApoE*$^{-/-}$ (g-n) mice administered a single 15-min light pulse (yellow bar) at circadian time (CT) 14-16 (early subjective night) in constant darkness (x-axis: hours; y-axis: days). Males are shown in a,b, and g-i. Females are shown in c-f and j-n.

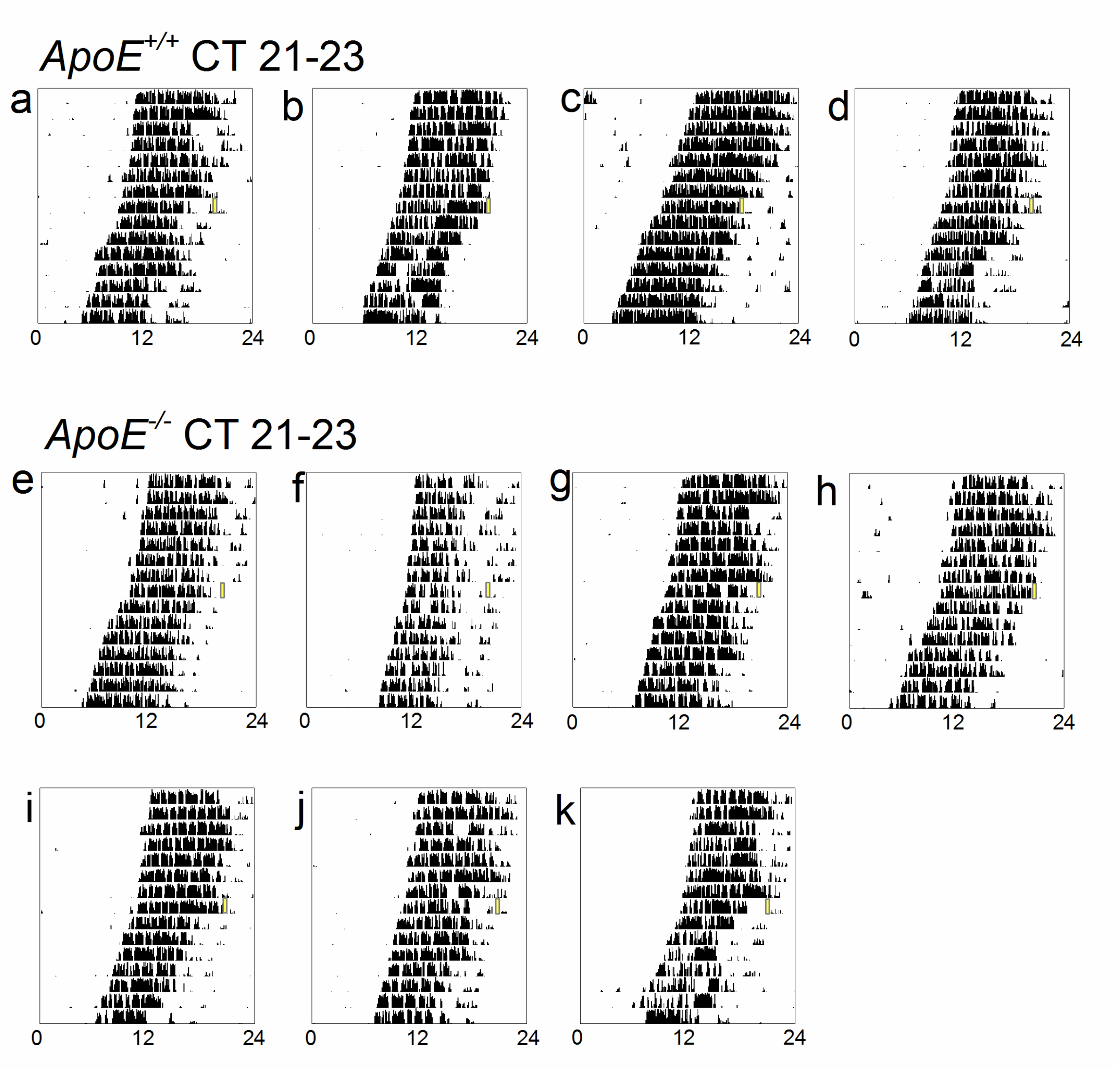

Figure S8. Phase responses to light pulses at CT 21-23 in *ApoE*$^{+/+}$ and *ApoE*$^{-/-}$ mice. Single-plotted actograms of *ApoE*$^{+/+}$ (a-d) and *ApoE*$^{-/-}$ (e-l) mice administered a single 15-min light pulse (yellow bar) at circadian time (CT) 21-23 (late subjective night) in constant darkness (x-axis: hours; y-axis: days). Males are shown in e and f. Females are shown in a-d and g-k.

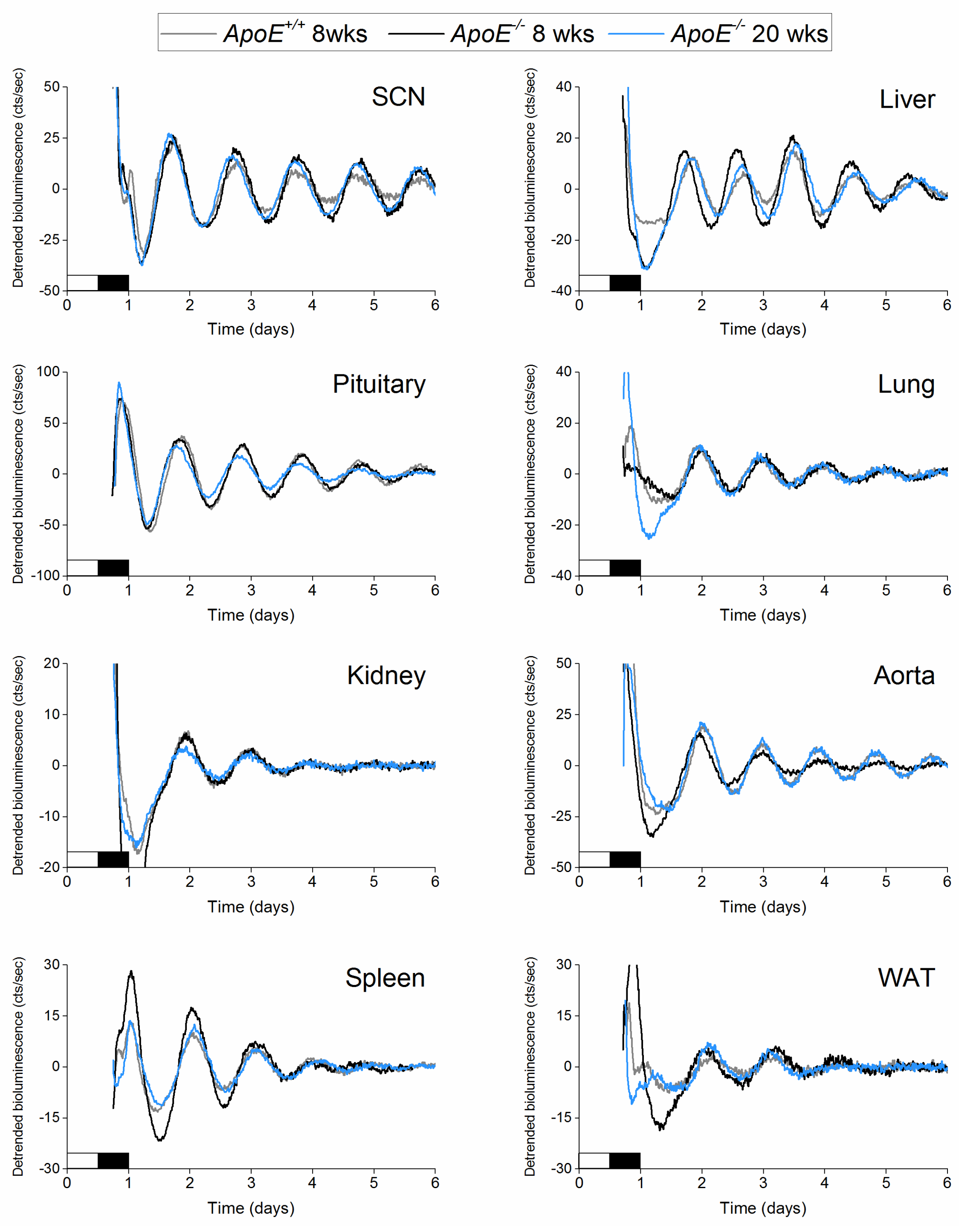

Figure S9. PERIOD2::LUCIFERASE bioluminescence rhythms in *ApoE*$^{+/+}$ and *ApoE*$^{-/-}$ tissues. Detrended bioluminescence recorded from tissue explants cultured from *ApoE*$^{+/+}$ at 8 weeks old (gray traces), *ApoE*$^{-/-}$ mice at 8 weeks old (black traces), and *ApoE*$^{-/-}$ mice at 20 weeks old (blue traces) that were heterozygous *PER2::LUC*. The lighting condition on the day the tissues were cultured is indicated for the first day; open bars are light and black bars are dark. The tissues were cultured during the 2h before lights out.

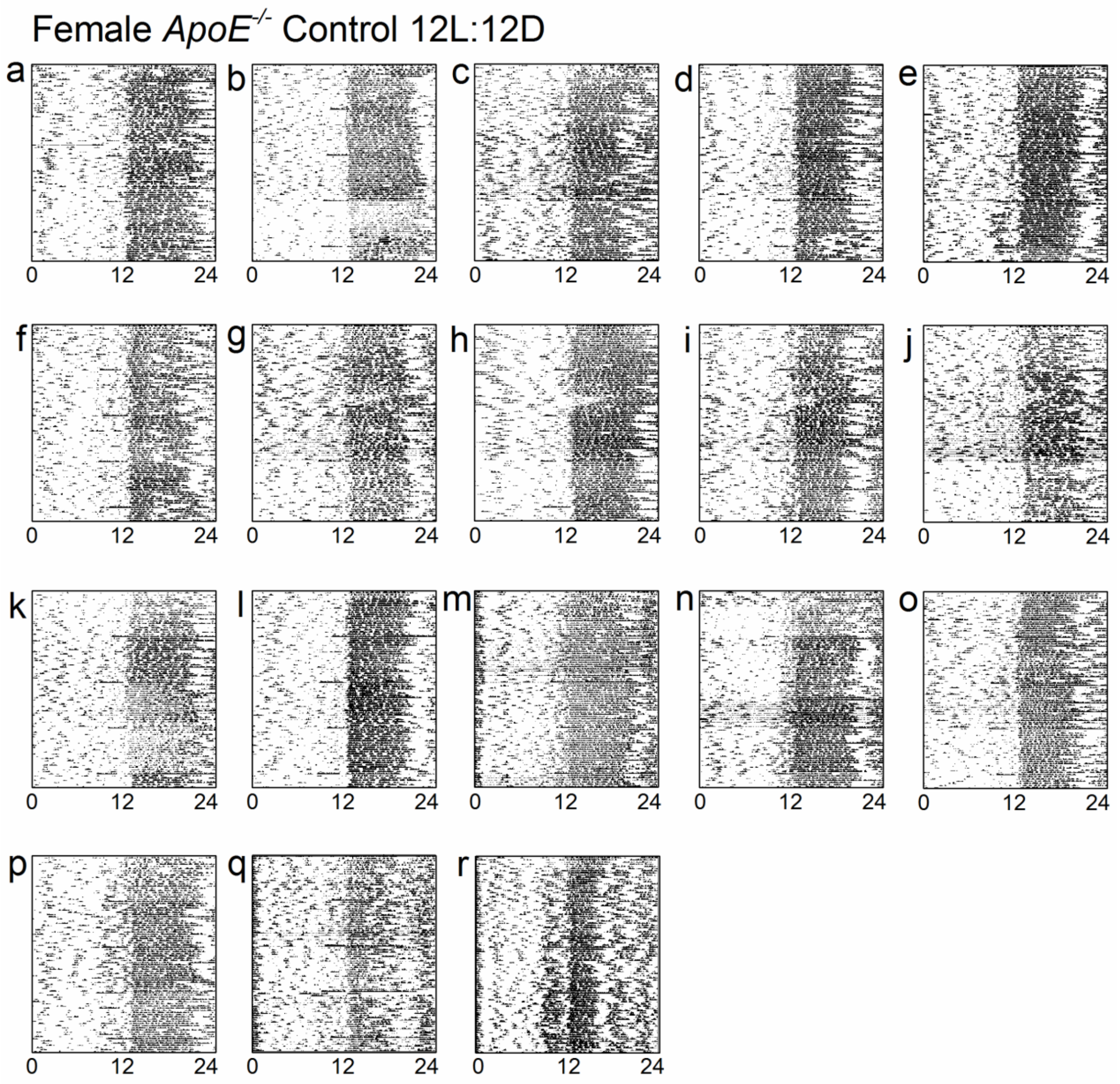

Figure S10. Locomotor activity of female *ApoE*$^{-/-}$ mice in 12L:12D. Single-plotted actograms of locomotor activity of individual female *ApoE*$^{-/-}$ mice (a-r) housed in control 12L:12D for 13 weeks (x-axis: hours; y-axis: days).

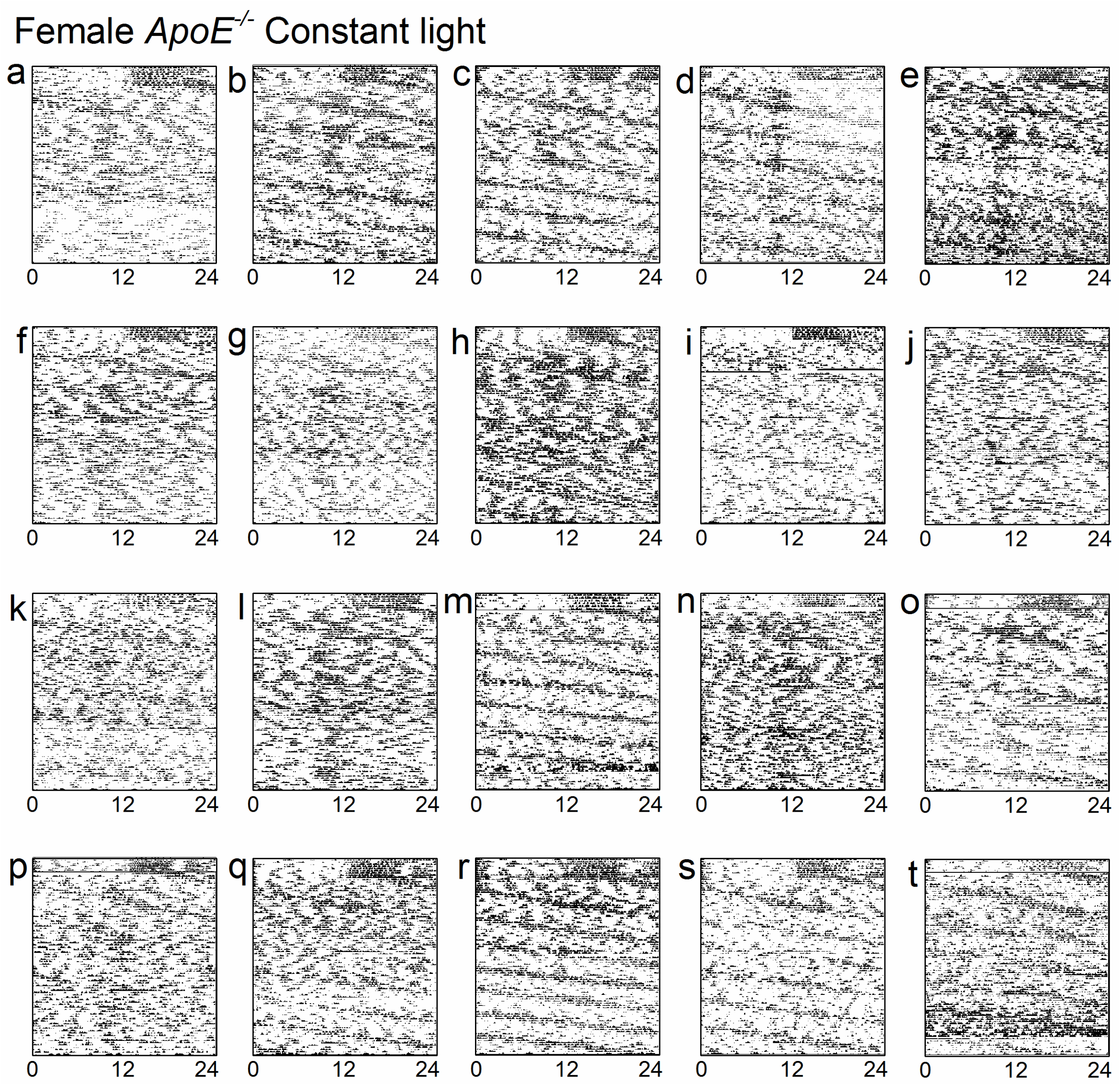

Figure S11. Locomotor activity of female *ApoE*$^{-/-}$ mice in constant light. Single-plotted actograms of locomotor activity of individual female *ApoE*$^{-/-}$ mice (a-u; x-axis: hours; y-axis: days). The mice were housed in 12L:12D (0h is lights on and 12h is lights off) for 1 week and then released into constant light for 12 weeks. One actogram was excluded due to a faulty infrared sensor.

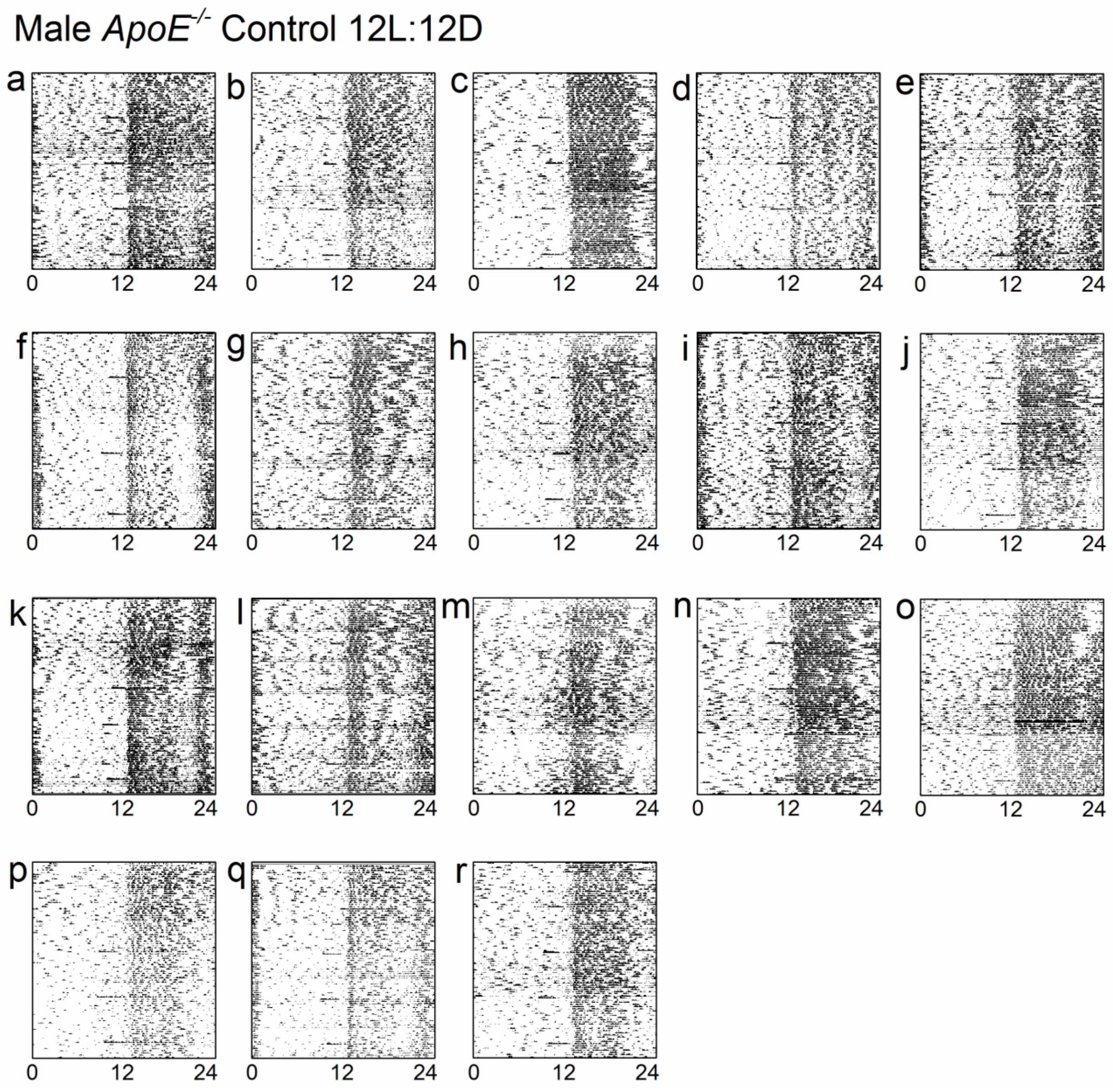

Figure S12. Locomotor activity of male *ApoE*$^{-/-}$ mice in 12L:12D. Single-plotted actograms of locomotor activity of individual male *ApoE*$^{-/-}$ mice (a-r) housed in control 12L:12D (0h is lights on and 12h is lights off) for 13 weeks (x-axis: hours; y-axis: days). One actogram was excluded due to a faulty infrared sensor.

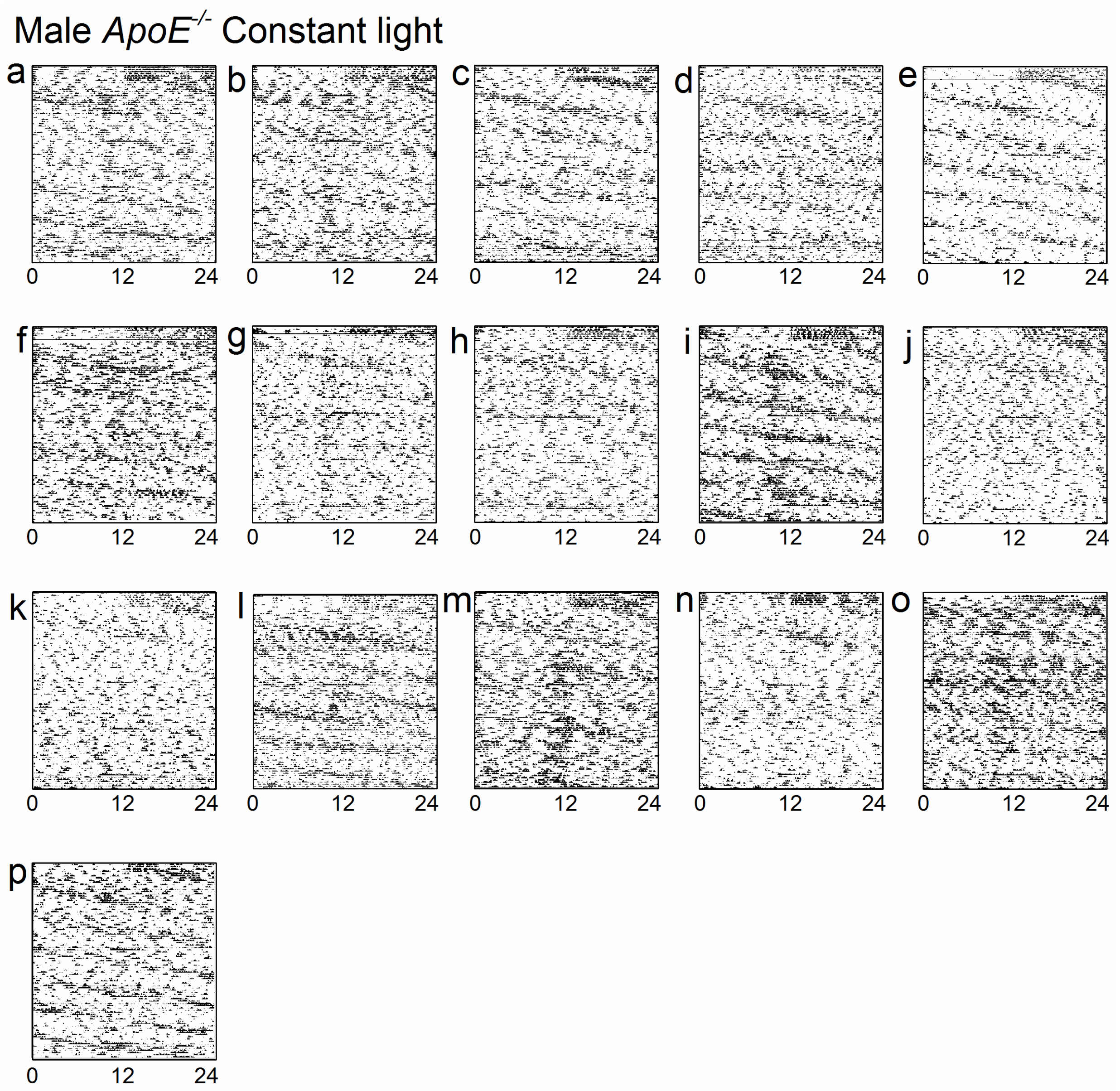

Figure S13. Locomotor activity of male *ApoE*$^{-/-}$ mice in constant light. Single-plotted actograms of locomotor activity of individual male *ApoE*$^{-/-}$ mice (a-p; x-axis: hours; y-axis: days). The mice were housed in 12L:12D (0 h is lights on and 12h is lights off) for 1 week and then released into constant light for 12 weeks.

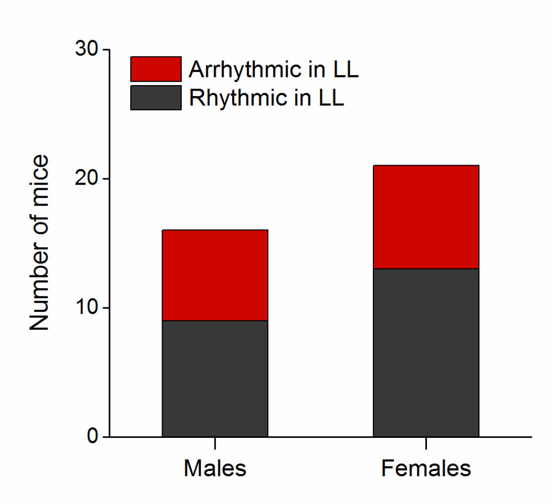

Figure S14. Chronic constant light (LL) exposure disrupts general locomotor activity rhythms in *ApoE*$^{-/-}$ mice. *ApoE*$^{-/-}$ mice were housed in LL for 12 weeks. χ^2 periodograms were used to determine whether general locomotor activity was rhythmic in the final 28 days in constant light. Activity was rhythmic if a dominant peak exceeded alpha set at p=0.001 (period range 20-36h).

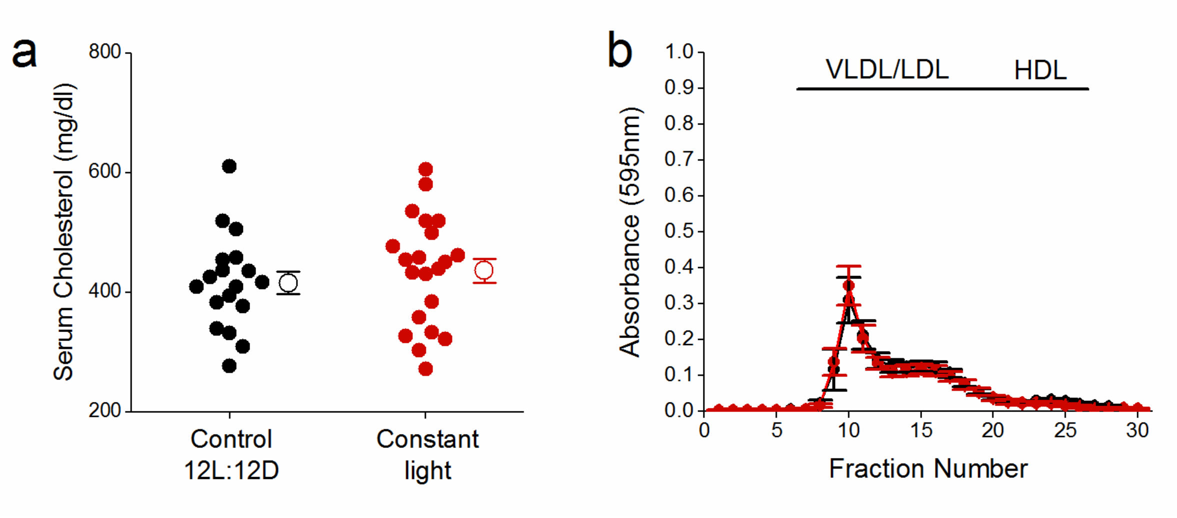

Figure S15. Constant light exposure does not alter cholesterol concentration and lipoprotein distribution in female *ApoE*$^{-/-}$ mice. Total serum cholesterol concentrations (a) and distribution of cholesterol on lipoproteins (b, mean ± SEM; FPLC was performed on n=4-5/group) of female *ApoE*$^{-/-}$ mice housed in control 12L:12D or constant light for 12 weeks. In a, data from individual mice are closed symbols and open symbols are mean ± SEM.

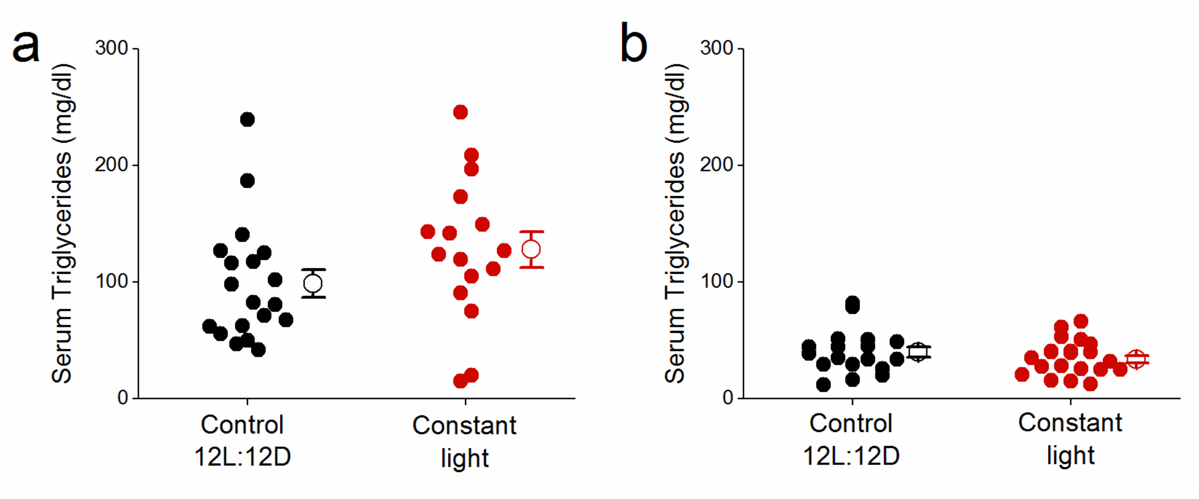

Figure S16. Constant light exposure does not alter serum triglyceride concentrations in male and female *ApoE*$^{-/-}$ mice. Total serum triglyceride concentrations of male (a) and female (b) *ApoE*$^{-/-}$ mice housed in control 12L:12D or constant light for 12 weeks. Data from individual mice are closed symbols and open symbols are mean ± SEM.

Supplementary Tables

Table S1. Descriptive statistics and statistical analyses of circadian behavior.

Circadian behavior parameter	*ApoE*$^{+/+}$ mean ± SEM (n)	*ApoE*$^{-/-}$ mean ± SEM (n)	Test*	Test statistic, *p* value
Activity in 12L:12D[#]				
Amplitude (Q_p)	1165 ± 42 (13)	1099 ± 29 (13)	*t*-test	t_{24}=1.29, *p*=0.21
Daily Activity (wheel rev x 10^3)	26.3±1.6 (13)	26.3±1.5 (13)	*t*-test	t_{24}=-0.002, *p*=0.99
Phase Angle of Entrainment (h)	0.34 ± 0.08 (13)	0.47 ± 0.09 (13)	Mann-Whitney	U=61, *p*=0.24
Activity in Constant Darkness^				
Period (h)	23.73 ± 0.03 (13)	23.80 ± 0.02 (13)	*t*-test	t_{24}=-1.85, *p*=0.08
Amplitude (Q_p)	1385 ± 34 (13)	1295 ± 22 (13)	*t*-test	t_{24}=2.24, *p*=0.03
Daily Activity (wheel rev x 10^3)	37.8 ± 1.7 (13)	32.9 ± 1.8 (13)	*t*-test	t_{24}=2.03, *p*=0.05
Activity in Constant Light[&]				
Period (h)	25.44 ±0.16 (5)	25.05 ± 0.20 (5)	Mann-Whitney	U=18, *p*=0.29
Amplitude (Q_p)	659 ± 99 (5)	570 ± 60 (5)	*t*-test	t_8=0.77, *p*=0.46
Daily Activity (wheel rev x 10^3)	9.9 ± 2.7 (5)	9.5 ± 2.0 (5)	*t*-test	t_8=0.11, *p*=0.92
Light Responsiveness				
Circadian time 8-10	-0.02± 0.23 (4)	0.01±0.15 (4)	*t*-test	t_6=-0.08, *p*=0.94
Circadian time 14-16	-1.95±0.24 (6)	-2.12±0.24 (8)	*t*-test	t_{12}=0.48, *p*=0.64
Circadian time 21-23	1.01±0.27 (4)	0.64±0.16 (7)	Mann-Whitney	U=22, *p*=0.16

*Tests are two-tailed; [#]7 days in 12L:12D; ^Days 1-7 in constant darkness; [&]Days 1-7 in constant light

Table S2. Circadian parameters of bioluminescence rhythms in *ex vivo* tissues.

Circadian parameter	Tissue	*ApoE*$^{+/+}$ 8 weeks old (n)	*ApoE*$^{-/-}$ 8 weeks old (n)	*ApoE*$^{-/-}$ 20 weeks old (n)	One-way ANOVA
**Period (h)**	SCN	24.1 ± 0.1 (11)	24.4 ± 0.1 (8)	24.3 ± 0.1 (5)	$F_{(2,21)}$= 2.6, p=0.10
	Liver	21.0 ± 0.4 (6)	21.1 ± 0.3 (7)	20.7 ± 0.1 (5)	$F_{(2,15)}$=0.27,p=0.76
	Pituitary	23.1 ± 0.2 (10)	23.4 ± 0.1 (8)	23.6 ± 0.3 (7)	$F_{(2,22)}$=1.48, p=0.25
	Lung	23.3 ± 0.3 (10)	23.3 ± 0.3 (5)	23.5 ± 0.2 (6)	$F_{(2,18)}$=0.12, p=0.88
	Kidney	25.1 ± 0.2 (10)	24.7 ± 0.2 (5)	25.0 ± 0.2 (7)	$F_{(2,19)}$=0.69, p=0.51
	Aorta	22.7 ± 0.3 (9)	23.4 ± 0.3 (5)	23.1 ± 0.4 (7)	$F_{(2,18)}$=1.03, p=0.38
	Spleen	23.6 ± 0.3 (9)	23.1 ± 0.5 (4)	23.2 ± 0.7 (5)	$F_{(2,15)}$=0.39, p=0.68
	WAT	26.2 ± 0.4 (6)	26.2 ± 0.2 (5)	26.6 ± 0.2 (3)	$F_{(2,11)}$=0.43, p=0.66
**Amplitude (counts/s)**	SCN	96.6 ± 6.6 (11)	91.5 ±1 9.4 (9)	96.5 ±15.2 (5)	$F_{(2,22)}$=0.10, p=0.90
	Liver	21.2 ± 3.8 (9)	19.3 ± 3.0 (10)	23.9 ± 5.1 (6)	$F_{(2,22)}$=0.33, p=0.72
	Pituitary	119.7 ± 13.7 (10)	124 ± 17.7 (8)	104.7 ± 15.2 (7)	$F_{(2,22)}$=0.38, p=0.69
	Lung	21.3 ± 6.7 (9)	25.0 ± 84.6 (6)	21.9 ± 4.3(6)	$F_{(2,18)}$= 0.17, p=0.84
	Kidney	6.1 ± 1.0 (8)	6.5 ± 1.6 (8)	6.4 ± 1.1 (7)	$F_{(2,20)}$= 0.02, p=0.98
	Aorta	28.3 ± 8.9 (9)	29.2 ± 5.3 (8)	21.1 ± 3.6 (6)	$F_{(2,20)}$= 0.34, p=0.71
	Spleen	20.7 ± 3.1 (9)	30.9 ± 5.6 (7)	21.9 ± 5.3 (6)	$F_{(2,19)}$=1.54, p=0.24
	WAT	11.27 ± 6.3 (6)	15.81±7.03 (3)	ND	t_8= 0.54, p=0.61$^{\#}$
**Phase (h after lights on)**	SCN	35.7 ± 0.4 (11)	35.7±0.3 (9)	36.3 ± 0.4 (6)	$F_{(2,23)})$=0.64, p=0.54
	Liver	37.5 ± 0.5 (11)	37.3 ± 0.4 (10)	37.9 ± 0.4 (5)	$F_{(2,23)}$=0.14, p=0.87
	Pituitary	38.6 ± 0.2 (10)	38.4 ± 0.3 (9)	37.9 ± 0.5 (7)	$F_{(2,23)}$=1.08, p=0.36
	Lung	40.4 ± 0.5 (12)	40.6 ± 0.4 (7)	41.9 ± 0.3 (6)	$F_{(2,22)}$=2.09, p=0.15
	Kidney	41.3 ± 0.3 (12)	41.3 ± 0.3 (9)	41.7 ± 0.4 (7)	$F_{(2,25)}$=0.54, p=0.59
	Aorta	42.8 ± 0.6 (10)	42.2 ± 0.4 (8)	42.9 ± 0.5 (7)	$F_{(2,22)}$=0.48, p=0.62
	Spleen	43.9 ± 0.5 (9)	43.3 ± 0.6 (7)	44.4 ± 0.5 (6)	$F_{(2,19)}$=0.94, p=0.41
	WAT	45.5 ± 0.8 (6)	45.5 ± 0.7 (7)	44.6 ± 0.7 (3)	$F_{(2,13)}$=0.33, p=0.73

Data are mean ± SEM
ND: not determined because goodness of fit was less than 90% for most samples in this group.
$^{\#}$Groups were compared using two-tailed Student's *t*-test

Table S3. Descriptive statistics and statistical analyses of atherosclerosis and lipids.

	**Control 12L:12D** mean ± SEM (n)	**Constant light** mean ± SEM (n)	**Test***	**Test statistic, *p* value**
En face lesion area (% of arch)				
Males	2.97 ± 0.39 (19)	5.67 ± 0.69 (16)	*t*-test	t_{33}=-3.56, p=0.001
Females	2.69 ± 0.41 (18)	3.61 ± 0.42 (21)	Mann-Whitney	U=127, p=0.08
Lesion area of aorta roots (mm^2)				
Males	0.05 ± 0.01 (9)	0.09 ± 0.02 (9)	Mann-Whitney	U=17, p=0.04
Females	0.10 ± 0.03 (7)	0.16 ± 0.03 (14)	*t*-test	t_{19}=-1.32, p=0.20
Total serum cholesterol (mg/dl)				
Males	629.0 ± 33.3 (19)	804.6 ± 52.9 (16)	Mann-Whitney	U=73, p = 0.009
Females	415.9 ± 18.8 (18)	436.1 ± 20.1 (21)	*t*-test	t_{37}=-0.73, p = 0.47
Total serum triglycerides (mg/dl)				
Males	98.5 ± 11.7 (19)	127.7 ± 15.5 (16)	Mann-Whitney	U=96, p=0.07
Females	39.8 ± 4.4 (18)	35.2 ± 3.2 (21)	*t*-test	t_{37}=0.86, p=0.40
Macrophages (% lesion area)				
Males	66.4 ± 8.3 (9)	72.0 ± 7.4 (9)	*t*-test	t_{16}=-0.51, p=0.62
Females	Not determined	Not determined	-	-

Comparisons were made within the same sex for each parameter; *Tests are two-tailed

Table S4. Descriptive statistics and statistical analyses of metabolic parameters.

Metabolic Parameter	**Control 12L:12D** mean ± SEM (n)	**Constant light** mean ± SEM (n)	Test*	**Test statistic, *p* value**
Body weight (beginning, g)				
Males	23.9 ± 0.6 (19)	24.9 ± 0.4 (16)	*t*-test	t_{33}=-1.56, *p*=0.13
Females	18.5 ± 0.3 (18)	18.5 ± 0.3 (21)	*t*-test	t_{37}=-0.03, *p*=0.98
Body weight (ending, g)				
Males	29.5 ± 0.9 (19)	31.6 ± 1.1 (16)	*t*-test	t_{33}=-1.53, *p*=0.14
Females	22.2 ± 0.3 (18)	23.6 ± 0.3 (21)	*t*-test	t_{37}=-2.95, *p*=0.005
Cumulative food intake (g)				
Males	287.1 ± 5.2 (19)	282.3 ± 5.0 (16)	*t*-test	t_{33}=0.66, *p*=0.52
Females	271.3 ± 3.7 (18)	247.6 ± 2.8 (21)	*t*-test	t_{37}=5.21, *p*<0.001
Cumulative activity (x10^3 cts)				
Males	181.6 ± 10.5 (18^)	137.5 ± 9.8 (16)	Mann-Whitney	U=221, *p*=0.008
Females	214.5 ± 7.6 (18)	161.3 ± 10.1 (20^)	Mann-Whitney	U=295, *p*<0.001

Comparisons were made within the same sex for each parameter;
*Tests are two-tailed;
^1 mouse was removed from the activity analysis due to faulty infrared sensor

Table S5. A priori power analyses to compute required sample sizes^

Parameter	Difference in means	Standard deviation*	Effect size	Required sample size	Actual sample size
Activity in 12L:12D*					
Amplitude (Q_p)	638	424	1.5	n=9/grp	n=13/grp
Phase Angle of Entrainment (h)*	0.47	0.31	1.5	n=8/grp	n=13/grp
Activity in Constant Darkness*					
Period (h)	0.3	0.21	1.5	n=9/grp	n=13/grp
Amplitude (Q_p)	638	424	1.5	n=9/grp	n=13/grp
Activity in Constant Light*					
Period (h)	0.57	0.38	1.5	n=9/grp	n=5/grp
Amplitude (Q_p)	115	78	1.5	n=9/grp	n=5/grp
Light Responsiveness*					
Circadian time 14-16 (h)	0.62	0.41	1.5	n=8/grp	n=6-8/grp
En face lesion area[#] (% of arch)	0.94	0.91	1.0	n=16/grp	n=16-21/grp

^Power analyses were performed with alpha=0.05 and power=0.80 for unpaired, two-tailed *t*-tests. The effect size used for each analysis is indicated.
*Means and standard deviations were from our previous studies of wheel-running activity and PER2::LUC tissue rhythms in C57BL/6J mice[1,2]
*Means and standard deviations were from our pilot studies in *ApoE*$^{-/-}$ mice fed 10% kcal low-fat diet for 12 weeks.

Supplemental References

1 Pendergast, J. S., Friday, R. C. & Yamazaki, S. Distinct functions of Period2 and Period3 in the mouse circadian system revealed by in vitro analysis. *PloS one* **5**, e8552 (2010).

2 Pendergast, J. S., Friday, R. C. & Yamazaki, S. Photic Entrainment of Period Mutant Mice is Predicted from Their Phase Response Curves. *The Journal of neuroscience : the official journal of the Society for Neuroscience* **30**, 12179-12184 (2010).
